# The role of the antigorite + brucite to olivine reaction in subducted serpentinites (Zermatt, Switzerland)

**DOI:** 10.1186/s00015-020-00368-0

**Published:** 2020-10-26

**Authors:** Elias D. Kempf, Jörg Hermann, Eric Reusser, Lukas P. Baumgartner, Pierre Lanari

**Affiliations:** 1 Institute of Geological Sciences, University of Bern, Baltzerstrasse 1+3, 3012 Bern, Switzerland; 2 Institute of Geochemistry and Petrology, ETH Zurich, Clausiusstrasse 25, 8092 Zurich, Switzerland; 3 Institute of Earth Sciences, University of Lausanne, Quartier UNIL-Mouline Batiment Géopolis 4885, 1015 Lausanne, Switzerland

**Keywords:** Antigorite + brucite reaction, Olivine, Magnetite, Fluid release, Serpentinites, Eclogite formation, Subduction

## Abstract

Metamorphic olivine formed by the reaction of antigorite + brucite is widespread in serpentinites that crop out in glacier-polished outcrops at the Unterer Theodulglacier, Zermatt. Olivine overgrows a relic magnetite mesh texture formed during ocean floor serpentinization. Serpentinization is associated with rodingitisation of mafic dykes. Metamorphic olivine coexists with magnetite, shows high Mg# of 94–97 and low trace element contents. A notable exception is 4 µg/g Boron (> 10 times primitive mantle), introduced during seafloor alteration and retained in metamorphic olivine. Olivine incorporated 100–140 µg/g H_2_O in Si-vacancies, providing evidence for low SiO_2_-activity imposed by brucite during olivine growth. No signs for hydrogen loss or major and minor element diffusional equilibration are observed. The occurrence of olivine in patches within the serpentinite mimics the former heterogeneous distribution of brucite, whereas the network of olivine-bearing veins and shear zones document the pathways of the escaping fluid produced by the olivine forming reaction. Relic Cr-spinels have a high Cr# of 0.5 and the serpentinites display little or no clinopyroxene, indicating that they derive from hydrated harzburgitic mantle that underwent significant melt depletion. The enrichment of Mg and depletion of Si results in the formation of brucite during seafloor alteration, a pre-requisite for later subduction-related olivine formation and fluid liberation. The comparison of calculated bulk rock brucite contents in the Zermatt-Saas with average IODP serpentinites suggests a large variation in fluid release during olivine formation. Between 3.4 and 7.2 wt% H_2_O is released depending on the magnetite content in fully serpentinized harzburgites (average oceanic serpentinites). Thermodynamic modelling indicates that the fluid release in Zermatt occurred between 480 °C and 550 °C at 2–2.5 GPa with the Mg# of olivine varying from 68 to 95. However, the majority of the fluid released from this reaction was produced within a narrow temperature field of < 30 °C, at higher pressures 2.5 GPa and temperatures 550–600 °C than commonly thought. Fluids derived from the antigorite + brucite reaction might thus trigger eclogite facies equilibration in associated metabasalts, meta-gabbros, meta-rodingites and meta-sediments in the area. This focused fluid release has the potential to trigger intermediate depths earthquakes at 60–80 km in subducted oceanic lithosphere.

## Introduction

Serpentinites are considered as the most important source for water transport in subduction zones (Scambelluri et al. 1995; Ulmer and Trommsdorff 1995). The fluid release upon break down reactions in serpentinites is thought to be responsible for island arc magmatism (Schmidt and Poli 1998), and has the potential to transfer water from hydrous minerals such as brucite and antigorite to nominally anhydrous minerals such as metamorphic olivine, likely relevant for the deep water cycle (Kempf and Hermann 2018; Padrón-Navarta and Hermann 2017). The high pressure antigorite breakdown reaction was first suggested at Cima di Gagnone in meta-peridotite lenses by Evans and Trommsdorff (1978). It has been extensively investigated in experimental studies (Bromiley and Pawley 2003; Padrón-Navarta et al. 2010; Ulmer and Trommsdorff 1995; Wunder and Schreyer 1997) but was first described so far in Cerro del Almirez, a unique location in Spain (Padrón-Navarta et al. 2011; Scambelluri et al. 2001; Trommsdorff et al. 1998). Padrón-Navarta et al. (2013) showed that during the antigorite breakdown (antigorite = olivine + enstatite + chlorite + water) 5 wt% of the total rock mass is released as H_2_O. Depending on the bulk rock composition, antigorite + brucite (antigorite + brucite = olivine + chlorite + water) and chlorite-out (chlorite + enstatite = olivine + garnet + water) reactions release between 2 and 3 wt% H_2_O.

While the influence of the terminal breakdown of antigorite on element recycling (Garrido et al. 2005; Harvey et al. 2014; Hermann et al. 2005; Marchesi et al. 2013; Ryan and Chauvel 2014; Savov et al. 2005; Scambelluri et al. 2014, 2019; Spandler and Pirard 2013), subduction zone earthquakes (Dobson et al. 2002; Ferrand et al. 2017; Gasc et al. 2017; Hacker et al. 2003; Jung and Green 2004; Padrón-Navarta et al. 2011; Peacock 2001) and tectonics (Gerya et al. 2002; Guillot et al. 2015) has been widely discussed in the literature, less attention has been paid to the antigorite + brucite reaction (Bloch et al. 2018; Kawahara et al. 2016; Kempf and Hermann 2018; Peretti et al. 1992; Peters et al. 2020; Scambelluri et al. 1991, 1995; Sippl et al. 2019; Spandler et al. 2011), which is frequently exposed in high-pressure ophiolites. Brucite formed during ocean floor serpentinization (Klein et al. 2014) is thought to be produced at low oxygen fugacity conditions (Frost 1985; Peretti et al. 1992). The petrologic importance of brucite in hydrated metaperidotites was first investigated in experimental studies by Bowen and Tuttle (1949). Hostetler et al. (1966) showed its presence in obducted partially serpentinized mantle rocks from circum-Pacific orogenic mountain belts. They pointed out that at first order, the brucite content is controlled by the modal ratio of olivine-orthopyroxene and thus of the Si/Mg ratio of the parent ultramafic rock. Nevertheless, the reaction antigorite + brucite is generally underrepresented in geodynamic models and often ignored (Hacker 2008; Hacker et al. 2003; van Keken et al. 2011). The identification of metamorphic olivine from this reaction is difficult since it often coexists with relic mantle olivine. However, major and minor element signatures were shown to be unique to this type of olivine (Kunugiza 1982; Nozaka 2003). Moreover, Kempf and Hermann (2018) showed that the presence of brucite enables formation of Si-vacancies in olivine where four hydrogens sit in a tetrahedral site. Therefore, FTIR spectroscopy of metamorphic olivine provides a unique fingerprint to individuate olivine that has formed from the antigorite + brucite reaction.

In this study, we investigated in detail an exceptional, glacier-polished outcrop of high-pressure serpentinite in the Zermatt-Saas unit (Switzerland). The outcrop escaped pervasive deformation during the Alpine subduction. We show that a wealth of features related to the hydration of peridotite at the ocean floor as well as the dehydration reaction of antigorite + brucite to olivine during subduction are preserved. This field-based study demonstrates the significance of the olivine-forming reaction for water recycling in subduction zones and its links to subduction related processes.

### Geological setting

**Fig. 1:**
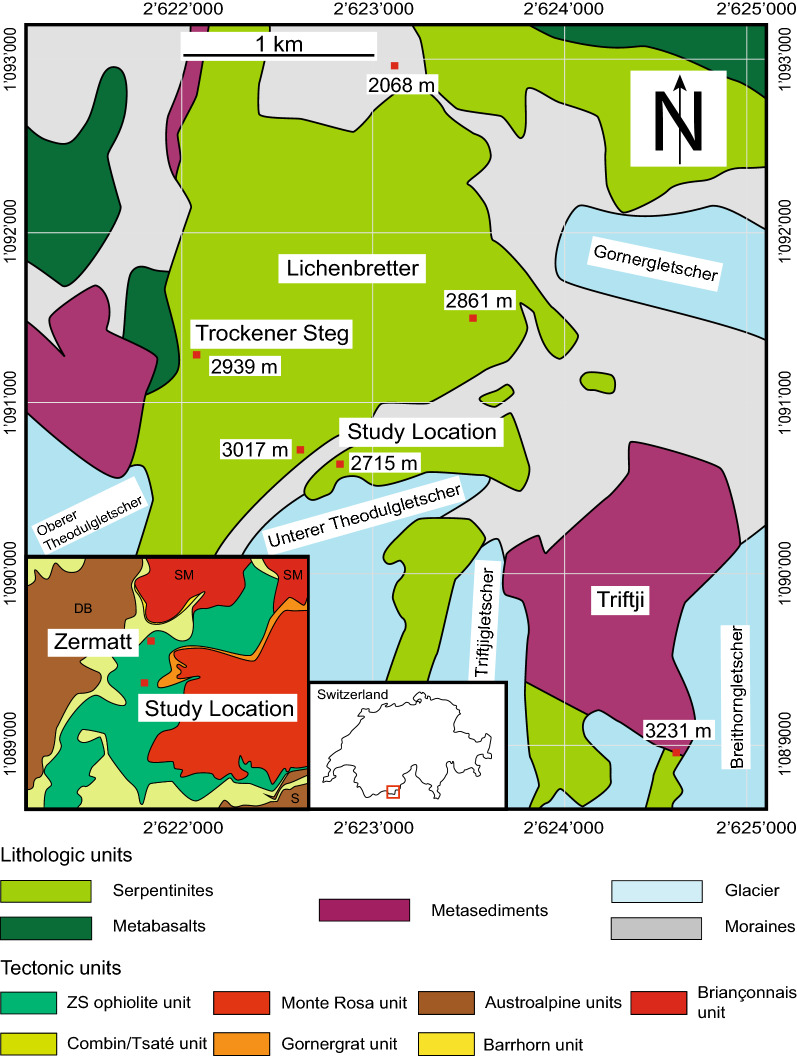
Geologic overview of the Zermatt-Saas ophiolite modified from (Bearth 1967; Li et al. 2004b; Seydoux and Baumgartner 2013; Weber and Bucher 2015). Study location: Coordinates in the map are given as CH1903 +/Lv95 Swiss coordinate grid. The study location lies between the point 2715 m.a.s.l. in the Swiss Geographical Survey map (1:25,000) and the WGS 84 coordinates (45.96572, 7.73498)

The Zermatt-Saas high pressure ophiolite belongs to the south Penninic nappe stack system in the western Alps at the border between Switzerland and Italy. It separates the overlying Austroalpine Dent Blanche nappe consisting of continental fragments from the Adriatic crust of the underlying Monte Rosa nappe representing basement fragments derived from the European continental margin (Escher and Beaumont 1997). The ophiolite is a remnant of the middle to late Jurassic (160–120 Ma) Piedmont-Ligurian oceanic lithosphere (Bill et al. 1997; Rubatto et al. 1998) in which the serpentinites represent a hydrated mantle section (Li et al. 2004b). Eclogite facies conditions of 2.2–2.5 GPa and temperatures between 540 and 610 °C are recorded mainly in eclogites associated to the serpentinites (Angiboust et al. 2009; Bearth 1967; Bucher et al. 2005; Bucher and Grapes 2009). The subduction of the oceanic unit occurred 41–49 Ma ago e.g. (de Meyer et al. 2014; Lapen et al. 2003; Rubatto et al. 1998; Skora et al. 2015). The unit was rapidly exhumed within 2–5 My manifested by a greenschist facies overprint at 400–500 °C and 0.4–0.6 GPa (Agard et al. 2009; Cartwright and Barnicoat 2002; de Meyer et al. 2014; Reinecke 1991; Rubatto et al. 1998; Skora et al. 2015). The serpentinite covers 30 km^2^ in the Zermatt area and shows massive and mylonite structures (Li et al. 2004b). Meta-mafic dykes within the serpentinites are abundant and most prominently represented by metarodingites (Li et al. 2004a; Li et al. 2008). Since the southern portion of the serpentinites in the Zermatt area are located at high altitudes (2300–4100 m.a.s.l.) large parts were covered by glaciers. During the past decades glacial retreat has uncovered new outcrops at higher altitudes. Their investigation is particularly instructive since the wall rock is smoothly abraded and, unlike most serpentinites in the area, shows hardly any surface weathering. This study focuses on an exceptional, ~ 100 × 100 m outcrop, close to the Unterer Theodulglacier that escaped mylonitic deformation (Fig. 1) and preserves a full orogenic cycle from mantle exhumation to seafloor hydration, subduction and exhumation.

## Analytical methods

**Fig. 2:**
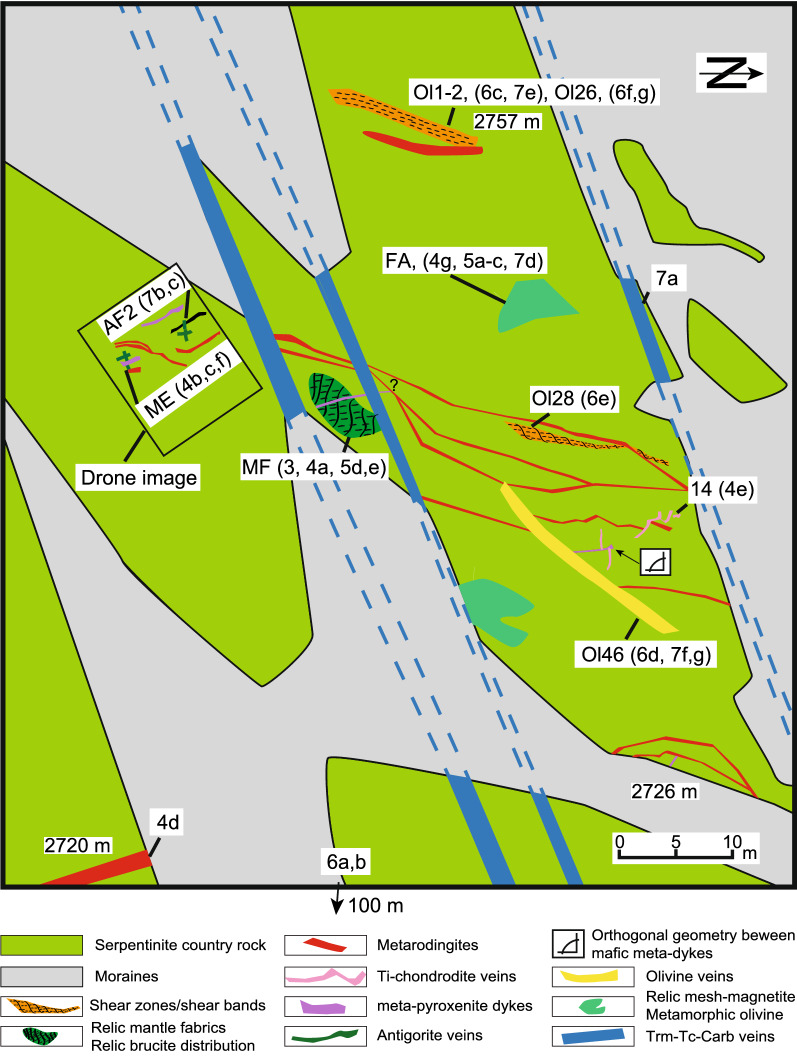
Simplified outcrop sketch of the investigated serpentinite body. Dashed lines are suggested traces of veins under the glacial deposits. The locations of samples and detailed figures are indicated (elevations are given in m.a.s.l.). A drone image of the outcrop indicated by the black rectangle is provided in Additional file 8: Figure S1

Polished thin sections (approximately 30 µm thickness for FA, MF, AF2, ME, Ol1, Ol2, Ol26, Ol46) were produced from representative samples and textures were investigated by optical microscopy, BSE imaging and quantitative WDS mapping. A subset of these samples were analysed also with LA-ICP-MS for trace elements. Fourier transform infrared (FTIR) spectroscopy measurements on olivine were obtained in 100–200 µm thick sections for H_2_O analyses. The locations of all samples are shown in Fig. 2 and the full sample list is presented in Additional file 1: Table S1.

### Electron probe micro analyser (EPMA)

A JEOL JXA-8200 Superprobe equipped with five wavelength dispersive crystal spectrometers (WDS) and one energy dispersive spectrometer (EDS) housed at the Institute of Geological Sciences, University of Bern was used for quantitative spot analyses and X-ray mapping. A backscatter detector (BSE) was used for imaging and micro-textural analyses. Samples were measured with 15 kV acceleration voltage and 20 nA probe current. Measurement time included 20 s on the peak and 10 s on background positions on either side of the peak. Natural and synthetic oxide and silicate minerals were used as standards. X-ray maps were acquired using a pixel size of 2 µm, a dwell time of 200 ms and a probe current of 100 nA. Spot analyses used as standards for the map quantification were acquired prior to mapping with the WDS spectrometers. Maps were processed, calibrated and analysed using the software XMapTools (Lanari et al. 2014, 2019).

### Laser ablation inductively coupled mass spectrometry (LA-ICP-MS)

Trace element analyses were measured by LA-ICP-MS using a Geolas Pro 193 nm ArF excimer laser with an ELAN DRC-e quadrupole ICP-MS. Optimization of the instrument followed the strategy protocol of Pettke et al. (2012). The mass spectrometer was optimised on NIST 610 and 612 glasses and the GSD-1G glass was used as an external standard. The laser fluence was set to 6 J·cm^−2^ with a repetition of 10 Hz. Spot diameters were chosen as large as possible between 60 and 120 µm. Background measurements lasted 50 s before each set whereas > 30 s measurement intervals were integrated and recalculated to element mass. MgO was used as an internal standard from quantitative EPMA analyses and the MATLAB©-based programme SILLS was used for data reduction (Guillong et al. 2008).

### Fourier transform infrared spectroscopy (FTIR)

For FTIR measurements, 200/100 µm thick sections were analysed. Only inclusion free olivine grains were measured with a MCT (Mercury-Cadmium-Telluride) detector. The water maps were measured using a FPA (Focal Plane Array) detector. A total of 4096 acquired spectra (for a detailed description see Data Directory of Kempf and Hermann (2018) were corrected for base-line and atmospheric water compensation using the Opus^®^ software. Spectra obtained by MCT and FPA were renormalized to 1 cm thickness. For single-spot measurements (MCT) thicknesses of the samples were determined with a probe (dial) indicator having an accuracy of 1 µm. FPA maps were thickness corrected with the integrated overtones (Shen et al. 2014) and the water contents were recalculated with the Bell et al. (2003) absorption coefficient. For a more detailed description see Kempf and Hermann (2018).

## Results

### Field relations and sample descriptions

All the samples were collected from a continuous outcrop within an area of 100 × 100 m on glacially polished serpentinites partially covered by glacial debris (moraine). The outcropping rocks were only weakly affected by Alpine deformation. In Fig. 2, the main lithological and structural elements are presented schematically. Various meta-dykes and vein sets show cross-cutting relationships that are visible at the map scale (Fig. 2). The location of the important samples and references to outcrop photos are also shown on the map. In the following, the main features are described chronologically based on mineralogy, cross-cutting and overprinting relationships.

Mineral abbreviations are only used in Figures: Brucite (Bc), Ti-clinohumite (Ti-cl), Ti-chondrodite (Ti-ch), Magnetite (Mt), Calcite (Cc), Andradite (Andr), Uvarovite (Uvar), Cr-magnetite (Cr–Mt), Tremolite (Trm), Talc (Tc), Chrysotile (Chr), Cr-spinel (Cr-spin), Perovskite (Prv), Sulphide (Sulph). All other mineral abbreviations follow Whitney and Evans (2010).

#### Evidence for relic mantle fabrics

**Fig. 3:**
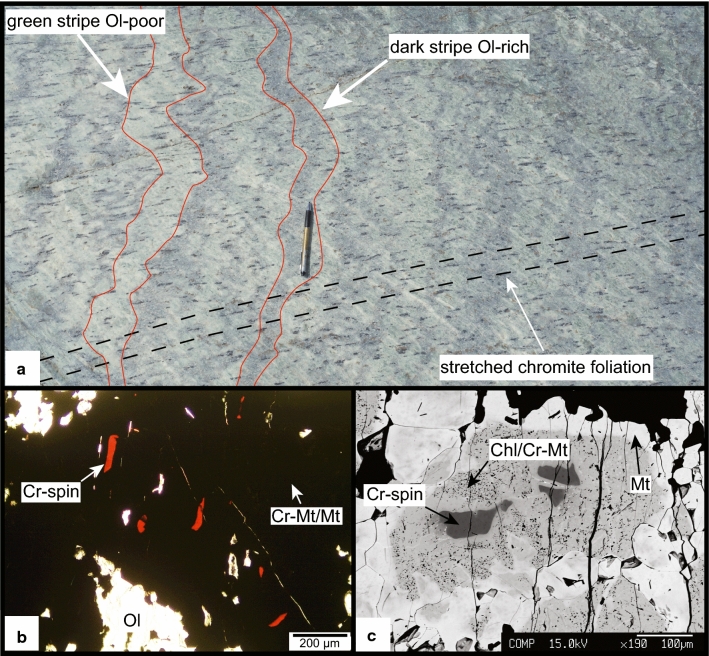
**a** Antigorite-olivine-chlorite serpentinite with large stretched chromites defining the pre-alpine foliation (samples MF and Ol28, see Fig. 2 for position, scale ~ 15 cm). **b** Polarised light image, with brown Cr-spinel relics in chromites (sample Ol28). **c** BSE image showing Cr-spinel core, Cr-magnetite and magnetite rims (sample Ol28)

In the serpentinite least affected by Alpine deformation (Fig. 3) several centimetre large and parallel oriented, stretched chromites are embedded in a statically grown antigorite-olivine matrix and are aligned along a preferred orientation constituting the pre-Alpine tectonite foliation (Fig. 3a). Occasionally, chromite contains three compositionally distinct zones consisting of brown Cr-spinel under polarised light, enclosed by Cr-magnetite rich in chlorite inclusion, and a magnetite-rich rim (Fig. 3b, c Table 1). The brown Cr-spinel is the only pre-oceanic relic phase observed in these rocks. No relic of mantle olivine was found. All olivines in these rocks are related to Alpine metamorphism, based on textures and composition (see below).

**Table 1:** Major and minor element composition of magnetite, Cr-magnetite, Cr-spinel

Sample	FA					Ol28				MF	Ol26
wt%	Polygon	Vein	Core	Core	Rim	Core	Core	Rim	Core	Polygon	Vein
TiO_2_	0.05	0.06	0.14	0.12	0.06	0.14	0.15	0.04	0.20	0.06	0.12
Al_2_O_3_	b.d.	0.15	0.27	0.03	0.03	4.38	0.23	0.01	26.82	0.09	0.02
Cr_2_O_3_	1.55	3.94	24.53	8.11	4.06	35.95	45.78	3.15	40.34	3.08	1.03
Fe_2_O_3_	68.81	67.14	46.33	63.07	66.40	31.90	27.39	67.10	4.50	67.80	69.10
FeO	24.60	23.49	15.62	21.62	24.04	15.93	15.87	25.61	10.51	23.07	24.07
MnO	0.31	0.58	2.77	0.82	0.45	2.16	2.86	0.23	1.45	0.44	0.46
NiO	0.32	0.29	0.24	0.37	0.23	0.19	0.06	0.33	0.06	0.64	0.43
MgO	3.70	4.47	7.92	5.50	4.07	9.24	8.64	3.16	15.89	4.06	3.96
CaO	b.d.	0.01	0.59	b.d.	0.01	b.d.	0.01	0.02	0.01	b.d.	b.d.
Total	99.35	100.15	99.01	99.71	99.36	99.93	101.05	99.68	99.79	99.45	99.28
Ti	0.002	0.002	0.004	0.003	0.002	< 0.001	0.040	< 0.001	< 0.001	0.002	0.003
Al	–	0.007	0.011	0.001	0.001	0.180	0.001	< 0.001	0.940	0.004	0.001
Cr	0.046	0.116	0.698	0.237	0.120	0.980	1.263	0.090	0.950	0.091	0.031
Fe^3^	1.950	1.873	1.254	1.751	1.873	0.830	0.719	1.900	0.100	1.909	1.955
Fe^2^	0.775	0.728	0.470	0.667	0.754	0.460	0.463	0.810	0.260	0.722	0.757
Mn	0.010	0.018	0.084	0.026	0.014	0.060	0.085	0.010	0.040	0.014	0.015
Ni	0.010	0.009	0.007	0.011	0.007	0.010	0.002	0.010	< 0.001	0.019	0.013
Mg	0.208	0.247	0.425	0.302	0.227	0.480	0.449	0.180	0.700	0.226	0.222
Ca	–	< 0.001	0.023	–	< 0.001	–	< 0.001	< 0.001	< 0.001	–	–
Mg#	20.7	24.7	43.1	30.1	22.7	47.5	42.2	17.8	70.0	23.1	22.0
Cr#	1.000	0.946	0.984	0.994	0.989	0.845	0.961	1.000	0.503	0.957	0.965

CaO and SiO_2_ mass fractions derive from secondary fluorescence and or beam deflection

Normalized to 3 cations and 4 oxygens Mg# = (Mg/(Mg + Fe + Ni + Mn))*100

Cr# = Cr/(Cr + Al)

*b.d.* below detection limit

#### Intrusion of mafic dykes

**Fig. 4:**
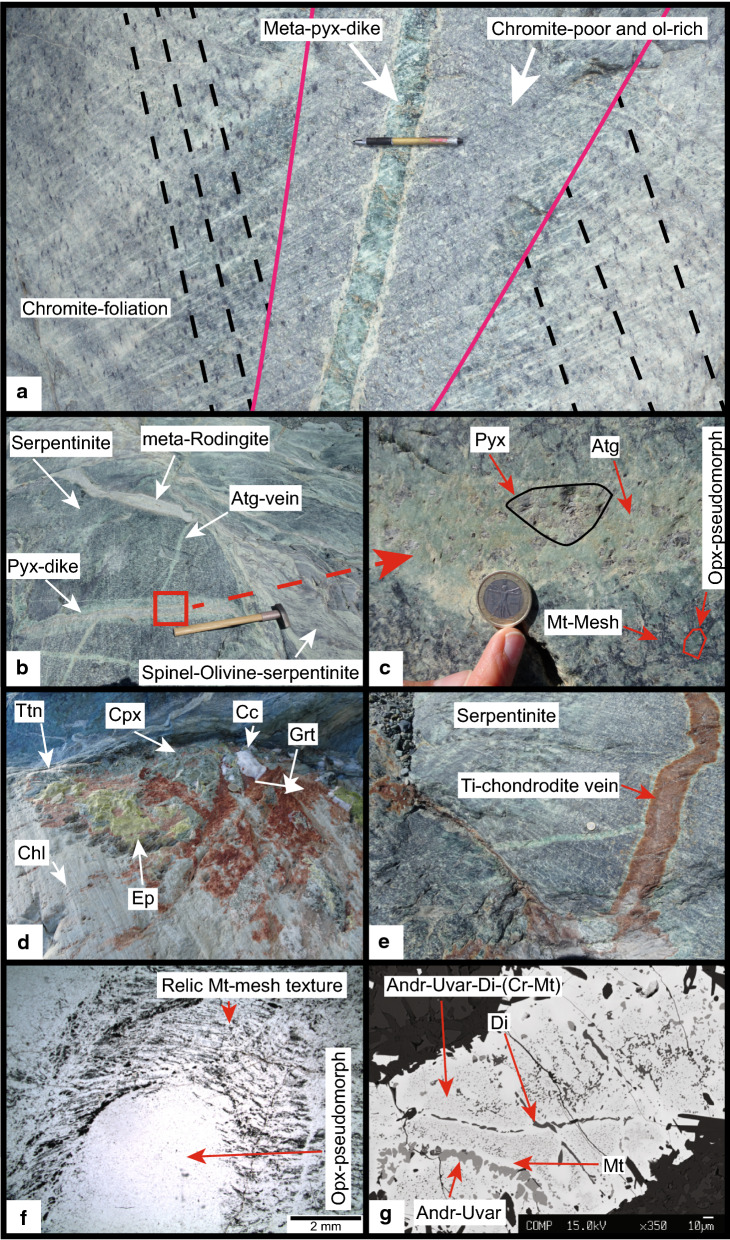
**a** Meta-mafic dyke generation_1_ crosscutting the chromite foliation with an olivine-rich and spinel-poor halo. **b** Serpentinite containing several meta-dyke (vein) generations (sample ME, see Fig. 2 for position) (Hammer scale length, ~ 60 cm). **c** Meta-pyroxenite dyke (vein) mostly serpentinized containing relic pyroxene (Euro coin 2.3 cm diameter). **d** Metarodingites showing the typical assemblage diopside, grossular and epidote in the core and chlorite at the rim together with titanite and calcite (Fig. 2). **e** Ti-chondrodite vein (meta-Ti-gabbro/basalt dyke) in serpentinite (sample 14). **f** Mt-mesh from serpentinized mantle olivine and antigorite pseudomorph after orthopyroxene (sample ME). **g** Andradite-uvarovite and diopside finely disseminated in partially replaced Cr-magnetite (sample FA)

The tectonite foliation (Fig. 4a) is crosscut by meta-pyroxenite dykes of several centimetre thickness and several meters up to tens of meters length (Fig. 4a, b), surrounded by an olivine and magnetite-rich but chromite poor halo. Occasionally, in the meta-dyke interiors relic pyroxenes are preserved (Fig. 4c). Most prominent are metarodingites consisting of epidote, grossular-rich garnet, diopside and titanite (Fig. 4d) that are tens of metres long and occasionally over one metre thick. The rodingites are enclosed by chlorite blackwalls with minor magnetite and rare perovskite. Less abundant red-brown veins consisting of Ti-chondrodite are nearly perpendicular to the metarodingites and meta-pyroxenites (Figs. 2, 4e). These veins and the rodingites are mutually crosscutting, i.e. in places the rodingite cross-cuts the Ti-rich veins and in other places the Ti-rich veins cross-cut the rodingite. Both cut the tectonite foliation and are boudinaged in areas with strong Alpine serpentine mylonitization. From this observation we conclude that the rodingite represents primitive mafic dykes whereas the Ti-chondrodite veins originate from more evolved Fe–Ti gabbroic or basaltic dykes (Gilio et al. 2019). The pervasive rodingitisation provides evidence for extensive fluid-rock interaction between the exhumed peridotites, mafic dykes and a seawater-derived fluid (Li et al. 2004a, b).

#### Oceanic serpentinization

**Fig. 5:**
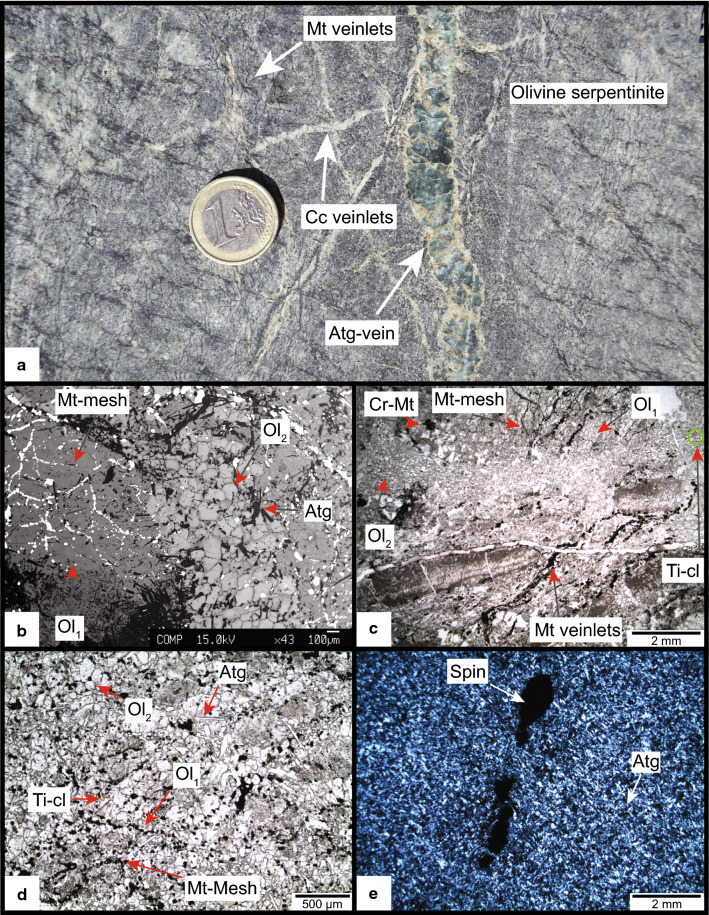
**a** Country rock sample FA (Fig. 2 for position) with grey-white olivine, black magnetite veinlets and cross-cutting antigorite and calcite veins (Euro coin 2.3 cm diameter). **b** Backscatter image of a large olivine_1_ enclosing the magnetite mesh texture. Small idioblastic olivines form the mosaic texture with antigorite. Note the partial or full disappearance of magnetite veins and mesh polygons in this domain (sample MF). **c** Thin section image (plain polarized light) of olivine_1_- and recrystallized olivine_2_ domains, note the absence of magnetite veins and mesh textures in domain_2_ (sample FA). **d** Plain polarised light image from a dark stripe consisting of large inclusion-rich olivine_1_ with magnetite polygons and idioblastic olivine_2_ forming a mosaic texture with antigorite (sample MF). **e** Crossed polarised light image of a green stripe (**a**) consisting of interlocked antigorite and relic chromites (sample MF)

The tectonite foliation and undeformed serpentinites are also crosscut by veinlets consisting of light to dark green serpentine associated with carbonates (Fig. 5a). Serpentinization of mantle olivine involves the formation of the characteristic mesh textures consisting of magnetite polygons filled with serpentine minerals and brucite (Evans 2004; O’Hanley 1996; Wicks and Whittaker 1977). In the least deformed rocks magnetite polygons are well preserved in both olivine-free and olivine-bearing serpentinites (Figs. 4f, 5b, c, d). The mesh texture is frequently accompanied with up to millimetre wide magnetite veins (Fig. 5c), commonly interpreted as conduits of aqueous fluid (Frost and Beard 2007). Antigorite domains with no magnetite mesh textures are interpreted to derive from former orthopyroxene (Fig. 4f). Hydration of mantle Cr-spinel (Fig. 3b, c) involves the formation of two distinct rims. The first consists of Cr-magnetite containing chlorite inclusions that are surrounded by later magnetite. During serpentinization, Ca metasomatism transformed the basaltic dykes into rodingites (Li et al. 2004a). In the serpentinites, Ca-mobility is manifested by the intergrowth of andradite and diopside with Cr-magnetite and magnetite (Fig. 4g). This association is well known in serpentinites and develops at very low oxygen fugacity together with FeNi-alloys (Frost 1985; Frost and Beard 2007).

#### Metamorphic olivine formation

Olivine is widespread in the serpentinites and appears either as darker shadings in very fresh outcrops where the glacier retreat was recent (Figs. 3a, 5a) or with an orange weathering colour in outcrops that have been exposed for a longer time. Olivine formation is best studied in sample FA (Fig. 5). Macroscopically, the FA rock is massive dark grey with sub-millimeter wide and several tens of centimetre long magnetite veins. The magnetite mesh texture and magnetite veins are overgrown by 0.5–2 cm large olivine_1_ (Fig. 5b, c, d) providing textural evidence for a metamorphic origin of the olivine postdating oceanic magnetite formation. This large olivine_1_ is partially recrystallized in sub-parallel bands (Fig. 5b, c) consisting of 50–200 micron sized polygonal olivine_2_ associated to Ti-clinohumite grains, forming a mosaic texture with antigorite and occasionally chlorite (Fig. 5b, c, d). In these mosaic domains, magnetite veins as well as the mesh texture are marginally offset and disappear (Fig. 5c). Opaque phases such as sulphides occur as inclusions in olivine_1_ and are absent in the mosaic domain (Fig. 5c, for sulphides see Additional file 2: Figure S2, see Piccoli et al. (2019)).

#### Shear zones and olivine veins

**Fig. 6:**
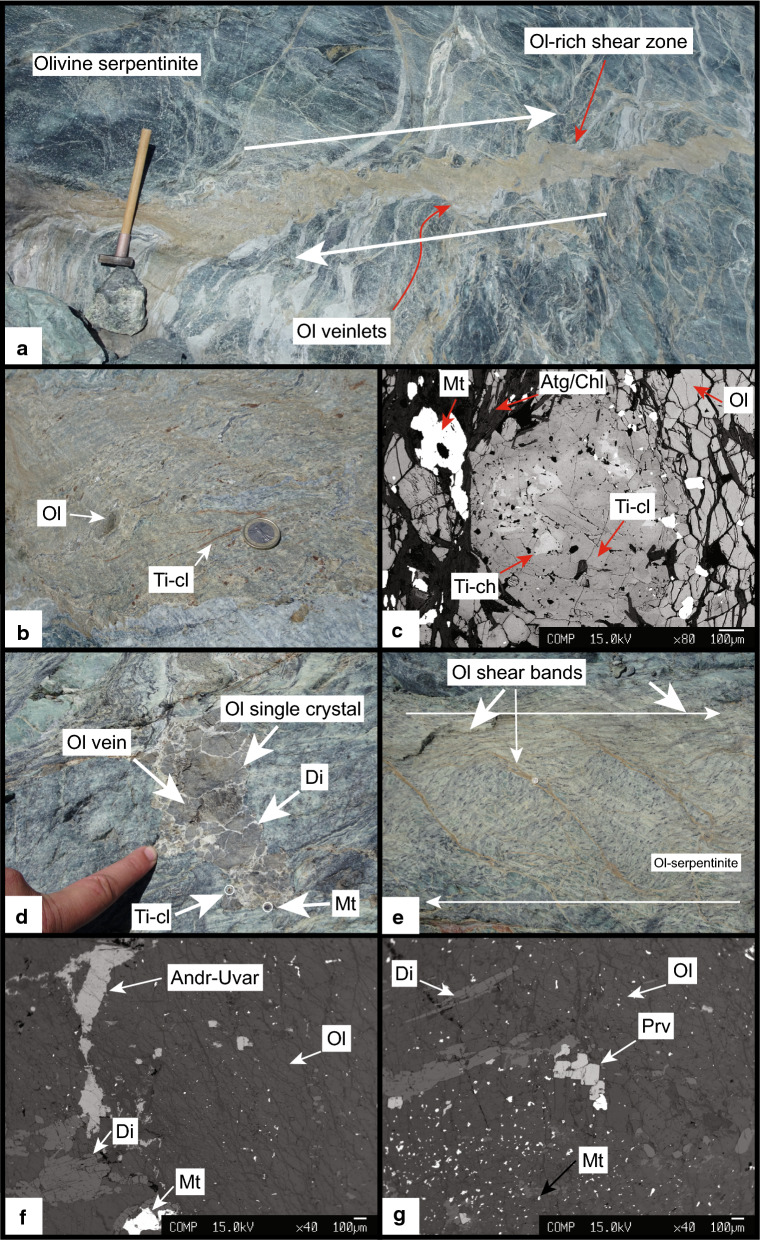
**a** Olivine-rich shear zone (Fig. 2 for position) with indicated sense of shear (hammer scale length: 60 cm). **b** Zoom-in of **a**, showing the presence of Ti-clinohumite in addition to olivine. **c** Ti-chondrodite grain replaced by Ti-clinohumite in an olivine-rich matrix (sample Ol1) (Euro coin 2.3 cm diameter). **d** Olivine vein consisting of centimetre sized single crystal olivines, interstitial diopside and red-brown Ti-clinohumite (Finger scale 8 cm). **e** Olivine shear bands, with indicated sense of shear. **f** Backscatter image of a large olivine showing the presence of andradite, diopside, magnetite and perovskite (**g**) (both sample Ol26)

Occasionally, sub-centimetre to several centimetre wide, olivine-rich veinlets of the olivine_2_-domain merge to form olivine-rich shear zones, which range from a few tens of centimetres to over 1 m thickness and are up to several tens of metres long (Fig. 6a). Idioblastic olivine grains show diameters of 100–500 microns and form a mosaic texture with variable amounts of chlorite, antigorite and Ti-clinohumite/Ti-chondrodite (Fig. 6b, c). Olivine veins are several centimetres wide and up to 1 m long. They often occur en-echelon in sheared domains (Figs. 2 and 6d, e) and contain up to several centimetre large single crystal olivines (Fig. 6d), few Ti-clinohumite grains and variable amounts of white diopside. Moreover, in an olivine vein juxtaposed to a metarodingite accessory andradite and perovskite (Fig. 6f, g) are present.

#### Retrograde features

**Fig. 7:**
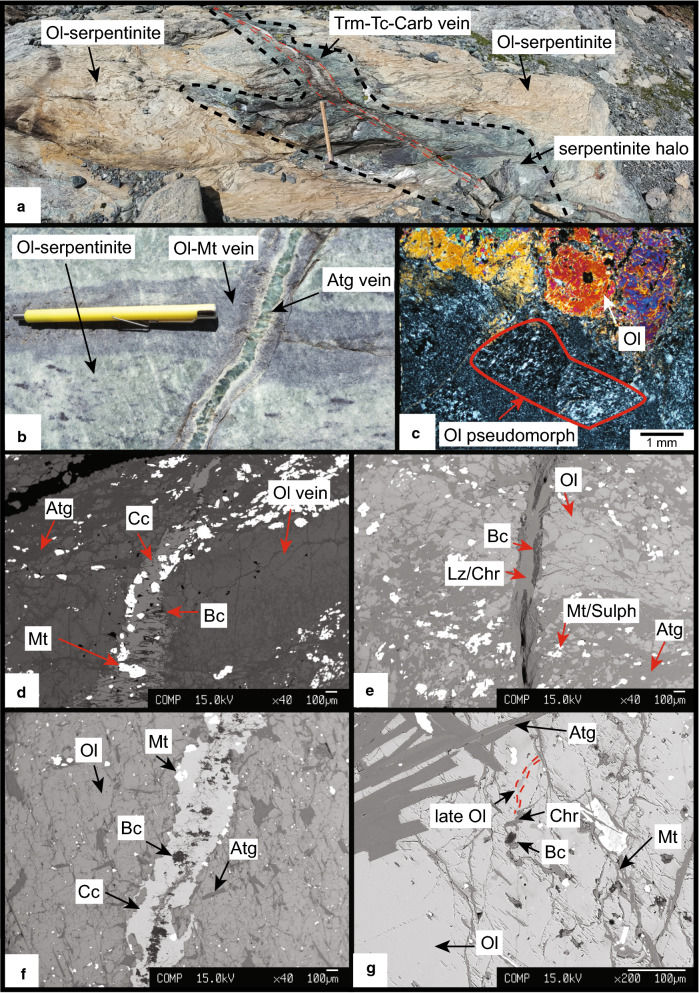
**a** Tremolite-talc-carbonate vein crosscutting olivine-rich country rock, note the serpentinization front (Fig. 2 for position). **b** Antigorite vein crosscutting an olivine-magnetite vein and the olivine-rich country rock (sample AF2). **c** Thin section image of **b** showing the pseudomorphic replacement of antigorite after olivine (sample AF2). **d** Late carbonate vein formation together with brucite, lizardite/chrysotile and magnetite (sample FA). **e** Brucite and lizardite/chrysotile veins crosscutting olivine in a shear zone (sample Ol1-2). **f** Calcite vein crosscutting a large olivine single crystal, note the coincidence of brucite and lizardite/chrysotile, magnetite-rich rims and low Mg# in the olivine in contact (sample Ol46). **g** Large blades of antigorite included by large olivine_2_ crystal which is partially replaced with higher Mg# olivine veinlets extending from veins with brucite and lizardite/chrysotile (sample Ol46)

Late tremolite-actinolite-talc-carbonate veins crosscut the prograde olivine-bearing serpentinite. The veins are several tens to hundreds of meters long and up to 2 m wide. Three large talc-magnesite veins are present in the centre of the outcrop (Fig. 2). The veins show a serpentinized (antigorite) halo at the contact to the olivine-rich-serpentinites (Fig. 7a). Also, 1–3 cm wide and up to several metres long antigorite veins crosscut the olivine-serpentinites and olivine-magnetite veins (Fig. 7b) pseudomorphically replacing olivine grains (Fig. 7c, sample AF2). Late brucite, lizardite/chrysotile (Chr/Lz), magnetite and sulphide minerals occasionally co-precipitated with calcite veins crosscut and replace prograde olivine (Fig. 7d (FA), e (Ol1-2), f, g (Ol46)). Additionally, olivines recrystallize to higher Mg# while magnetite co-precipitates (Fig. 7g and Table 2).

**Table 2:** Major and minor element composition of olivine

Sample	Ol1	Ol2	FA			MF			Ol28	Ol26		Ol46			AF2		
Generation	2	2	2	1	1	2	1	1	2	2	1	1	1	1	2	1	1
wt%	Core	Core	Core	Core	Rim	Core	Core	Rim	Core	Core	Core	Core	Alt. vein	Alt. rim	Core	Core	Rim
SiO_2_	41.59	41.43	42.05	42.31	42.61	41.50	41.17	42.14	41.90	41.49	41.62	41.79	41.84	41.69	41.95	42.21	42.59
TiO_2_	b.d.	b.d.	0.02	0.07	0.01	b.d.	b.d.	b.d.	0.02	b.d.	0.05	b.d.	0.02	0.02	0.01	0.01	0.04
Al_2_O_3_	b.d.	0.03	0.08	0.01	0.02	0.01	0.04	0.07	b.d.	b.d.	0.02	b.d.	b.d.	0.01	0.01	0.02	b.d.
Cr_2_O_3_	0.01	b.d.	0.01	0.02	0.03	0.01	b.d.	0.01	0.01	b.d.	b.d.	b.d.	b.d.	0.01	b.d.	0.01	0.01
FeO	4.66	4.60	4.58	4.21	2.40	5.34	5.23	2.15	4.58	4.63	4.72	5.40	2.47	3.93	5.25	4.64	2.25
MnO	0.29	0.24	0.20	0.20	0.22	0.21	0.21	0.23	0.16	0.27	0.31	0.36	0.20	0.18	0.23	0.23	0.28
NiO	0.16	0.15	0.18	0.22	0.13	0.23	0.17	0.15	0.17	0.15	0.16	0.22	0.11	0.17	0.27	0.21	0.19
MgO	53.30	53.64	53.68	53.99	54.90	52.97	53.06	54.85	53.48	53.07	52.84	52.95	55.46	54.04	53.19	53.10	55.53
CaO	b.d.	b.d.	0.02	0.01	b.d.	b.d.	0.01	b.d.	0.03	0.01	0.01	b.d.	b.d.	0.01	0.01	0.01	b.d.
Total	100.01	100.10	100.82	101.04	100.32	100.28	99.90	99.60	100.34	99.63	99.73	100.72	100.15	100.07	100.92	100.45	100.88
Si	0.997	0.992	0.999	1.001	1.006	0.995	0.991	1.002	1.000	0.998	1.000	0.998	0.992	0.995	0.999	1.006	1.000
Ti	–	–	< 0.001	0.001	< 0.001	–	–	–	< 0.001	–	< 0.001	–	< 0.001	< 0.001	< 0.001	< 0.001	0.001
Al	–	0.001	0.002	< 0.001	0.001	< 0.001	0.001	0.002	–	–	< 0.001	–	–	<0.001	< 0.001	0.001	–
Cr	< 0.001	–	< 0.001	< 0.001	0.001	< 0.001	–	< 0.001	< 0.001	–	–	–	–	< 0.001	–	< 0.001	< 0.001
Fe^+2^	0.093	0.092	0.091	0.083	0.047	0.107	0.105	0.043	0.091	0.093	0.095	0.108	0.049	0.079	0.105	0.093	0.044
Mn	0.006	0.005	0.004	0.004	0.004	0.004	0.004	0.005	0.003	0.006	0.006	0.007	0.004	0.004	0.005	0.005	0.006
Ni	0.003	0.003	0.003	0.004	0.003	0.004	0.003	0.003	0.003	0.003	0.003	0.004	0.002	0.003	0.005	0.004	0.004
Mg	1.904	1.915	1.900	1.904	1.932	1.893	1.904	1.943	1.902	1.902	1.893	1.885	1.960	1.923	1.888	1.886	1.944
Ca	–	–	< 0.001	< 0.001	–	–	< 0.001	–	0.001	< 0.001	< 0.001	–	–	<0.001	< 0.001	< 0.001	–
Mg#	94.9	95.0	95.1	95.4	97.3	94.2	94.4	97.5	95.1	94.9	94.8	94.0	97.3	95.7	94.3	94.9	97.3
Cat#	3.003	3.008	3.000	2.998	2.993	3.005	3.009	2.997	3.000	3.003	2.999	3.002	3.009	3.004	3.001	2.994	2.999

Alt. Vein = alteration vein (Fig. 7g, late ol)

Normalized to 4 oxygens

Cat# = number of cations

Mg# = (Mg/(Mg + Fe + Ni + Mn))*100

*b.d.* below detection limit

#### Summary of the main stages derived from field observations

The single outcrop preserves a complete history of extension and convergence related to the formation, subduction and exhumation of oceanic lithosphere during the Alpine orogeny. Mantle exhumation is documented by chromite in a tectonite foliation followed by intrusions of mafic dykes close to the ocean floor. Oceanic hydrothermal alteration produced a pervasive serpentinization coupled with magnetite formation and rodingitisation of mafic dykes. Alpine subduction, prograde metamorphism, and limited deformation is best documented in metamorphic olivine that occurs either statically in undeformed parts or dynamically in shear zones and en-echelon veins. Late, crosscutting veins are related to the exhumation of the rocks.

### Mineral compositions

**Fig. 8:**
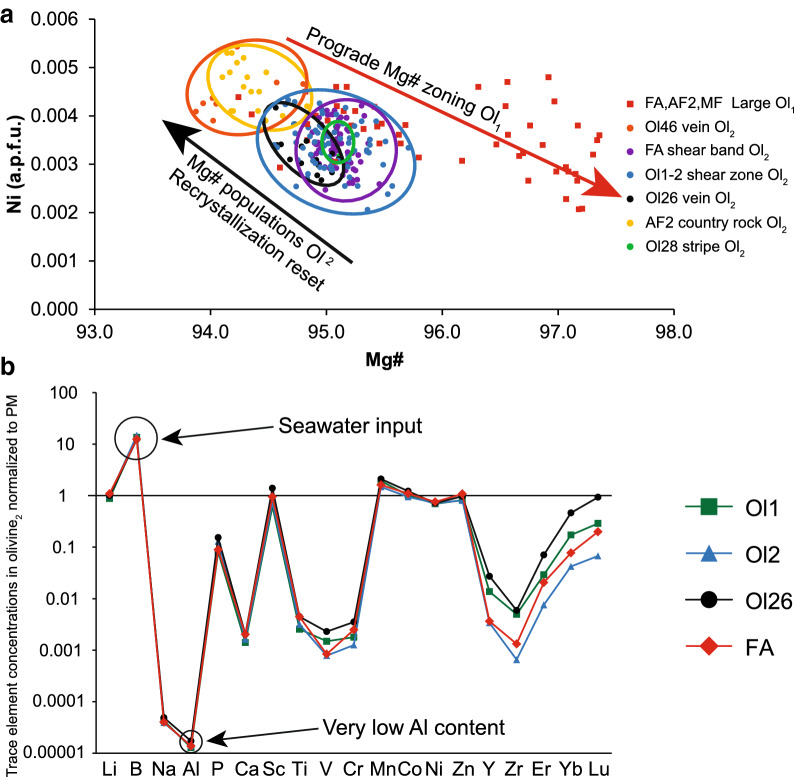
**a** Ni vs Mg# values indicate different populations of both olivine generations in Zermatt (Table 2). **b** Trace element contents of olivine_2_ generation normalized to primitive mantle compositions from McDonough and Sun (1995)

Major and minor element compositions from the three most important localities of the olivine forming reactions i.e., the country rock (MF, Ol28, FA, AF2), a shear zone (Ol1-2) and olivine veins (Ol26, Ol46) are reported in Table 2 and Fig. 8a. Trace elements in olivine are documented in Table 3 and Fig. 8b. Major and minor element compositions of antigorite and chlorite are given in Table 4 and of Ti-chondrodite and Ti-clinohumite in Table 5. Data from minor and accessory phases such as, andradite-uvarovite garnet, ilmenite, perovskite, brucite, lizardite/chrysotile and clinopyroxene are presented in Table 6. The full data set for all phase compositions used in this paragraph is given in Additional file 3: Table S2.

**Table 3:** Trace element composition of olivine_2_ generation

Samples	^1^n	Li	B	Na	Al	P	Ca	Sc	Ti	V	Cr	Mn	Co	Ni	Zn	Y	Zr	Er	Yb	Lu
Ol1	8	1.41	4.07	0.11	0.30	7.24	35.67	10.04	3.09	0.12	4.69	2008.02	106.61	1356.72	45.17	0.06	0.05	0.01	0.08	0.02
	^2^σ	0.33	0.46	0.05	0.03	1.70	4.16	2.91	0.93	0.08	3.31	117.99	5.07	124.56	2.63	0.02	0.01	<0.01	0.02	0.01
Ol2	8	1.61	4.42	0.10	0.33	11.60	41.24	12.09	3.82	0.06	3.30	1542.24	99.09	1407.91	44.29	0.01	0.01	b.d	0.02	b.d
	σ	0.27	0.62	0.02	0.03	4.66	7.49	2.89	3.41	0.02	1.32	170.70	6.94	187.96	5.29	< 0.01	< 0.01	–	0.02	–
Ol26	7	1.60	3.69	0.13	0.41	13.83	51.40	22.61	5.39	0.19	9.25	2198.17	128.38	1398.61	53.27	0.12	0.06	0.03	0.20	0.06
	σ	1.30	0.41	0.02	0.02	2.32	6.18	11.63	2.79	0.11	7.73	46.52	3.41	44.47	4.19	0.05	0.01	0.01	0.10	0.03
FA	5	1.74	3.73	0.11	0.32	8.02	51.17	15.38	5.35	0.07	6.53	1684.72	114.92	1479.36	59.33	0.02	0.01	0.01	0.03	0.01
	σ	0.55	0.90	0.04	0.05	2.09	11.12	7.71	2.29	0.03	3.43	38.69	3.08	30.89	4.47	< 0.01	< 0.01	< 0.01	0.01	< 0.01

Trace element mass fractions are presented in µg/g

^1^n number of grains analysed, ^2^σ standard deviation, b.d. below detection limit

**Table 4:** Major and minor element composition of antigorite and chlorite

	Antigorite												Chlorite		
Sample	FA						MF		AF2		Ol46		FA	Ol1	Ol46
wt%	p	p	p	p	p	r	p	r	p	r	p	r	p	p	p
SiO_2_	41.89	42.04	41.65	41.92	42.14	43.78	43.01	44.05	42.30	43.62	42.32	43.60	34.35	34.52	33.89
TiO_2_	0.02	0.01	0.02	0.02	b.d.	b.d.	0.02	0.01	b.d.	0.01	0.01	0.01	b.d.	b.d.	b.d.
Al_2_O_3_	2.53	2.59	2.56	2.33	2.77	1.30	2.18	0.78	2.15	1.39	2.58	1.30	11.55	12.59	11.49
Cr_2_O_3_	0.69	0.70	0.76	1.09	0.85	0.08	0.50	0.24	1.70	0.12	0.81	0.20	2.03	0.66	2.64
FeO	2.30	1.95	1.82	1.66	1.90	1.59	2.04	1.33	1.58	1.58	2.06	1.92	2.90	3.10	3.24
MnO	0.03	0.03	0.05	0.03	0.04	0.02	0.04	0.03	0.04	0.01	0.03	0.03	0.04	0.02	0.06
NiO	0.04	0.05	0.01	0.03	b.d.	0.08	0.16	0.06	0.08	0.09	0.03	0.02	0.10	0.07	0.10
MgO	39.53	40.53	40.69	39.94	40.04	40.66	40.33	41.78	39.89	40.58	39.98	40.30	35.92	35.51	35.45
CaO	b.d.	b.d.	0.02	b.d.	0.03	0.01	0.01	b.d.	0.02	0.01	b.d.	0.01	0.05	0.01	0.04
H_2_O	12.02	12.15	12.10	12.04	12.14	12.18	12.22	12.28	12.12	12.16	12.14	12.14	12.65	12.65	12.59
Total	99.05	100.04	99.68	99.06	99.90	99.71	100.53	100.56	99.87	99.59	99.94	99.51	99.58	99.13	99.49
Si	32.395	32.168	31.997	32.362	32.261	33.413	32.716	33.347	32.430	33.339	32.400	33.392	3.257	3.272	3.229
Ti	0.011	0.003	0.012	0.012	–	–	0.010	0.007	–	0.008	0.006	0.004	–	–	–
Al	2.306	2.336	2.318	2.120	2.497	1.169	1.957	0.695	1.943	1.251	2.325	1.170	1.291	1.406	1.290
Cr	0.424	0.424	0.462	0.666	0.514	0.052	0.300	0.141	1.030	0.073	0.489	0.122	0.152	0.050	0.199
Fe^+2^	1.488	1.248	1.170	1.072	1.216	1.012	1.298	0.842	1.013	1.010	1.319	1.229	0.230	0.246	0.258
Mn	0.022	0.021	0.033	0.019	0.026	0.013	0.026	0.022	0.025	0.008	0.019	0.017	0.003	0.001	0.005
Ni	0.025	0.028	0.006	0.019	–	0.047	0.098	0.040	0.049	0.058	0.017	0.010	0.008	0.005	0.008
Mg	45.560	46.220	46.588	45.964	45.695	46.260	45.716	47.137	45.580	46.235	45.612	46.010	5.076	5.017	5.034
Ca	–	–	0.016	–	0.024	0.010	0.005	–	0.013	0.009	–	0.005	0.005	0.001	0.004
H	62.000	62.000	62.000	62.000	62.000	62.000	62.000	62.000	62.000	62.000	62.000	62.000	8.000	8.000	8.000
Mg#	96.7	97.3	97.5	97.6	97.4	97.7	97.0	98.1	97.7	97.7	97.1	97.3	95.5	95.2	94.9
Cat#	82.230	82.449	82.601	82.233	82.233	81.976	82.125	82.229	82.083	81.991	82.187	81.958	10.022	10.001	10.027
Al#	0.29	0.29	0.29	0.26	0.31	0.15	0.24	0.09	0.24	0.16	0.29	0.15			
(Al + Cr)#	0.34	0.35	0.35	0.35	0.38	0.15	0.28	0.10	0.37	0.17	0.35	0.16			

*p* peak metamorphic core, *r* retrograde, *b.d.* below detection limit

Antigorite normalized to 147 oxygens and 62 (OH), Chlorite to 18 oxygens and 8 (OH), total iron as FeO

Cat# = number of cations

Mg# = (Mg/(Mg + Fe + Ni + Mn))*100

Al# = Al/8, (Al + Cr)# = (Al + Cr)/8, according to Padrón-Navarta et al. (2013)

**Table 5:** Major and minor element composition of Ti-chondrodite and Ti-clinohumite

	Ti-chondrodite				Ti-clinohumite								
Sample	14			Ol1	14		FA	AF2	Ol1		MF	Ol26	Ol46
wt%	l	l	s	s	r	r	p	p	p	p	p	p	p
SiO_2_	32.95	33.14	33.66	33.68	37.48	37.26	37.76	37.39	37.26	37.48	38.53	37.27	37.85
TiO_2_	8.35	9.14	7.61	8.71	3.37	3.72	3.16	2.13	4.88	3.37	2.50	2.98	3.77
Al_2_O_3_	b.d.	0.03	0.01	0.01	b.d.	0.02	b.d.	0.01	0.14	b.d.	0.01	0.03	0.01
Cr_2_O_3_	0.01	0.02	0.01	b.d.	b.d.	b.d.	0.01	0.03	0.01	b.d.	0.01	b.d.	0.01
FeO	7.08	6.23	5.42	3.38	4.81	3.42	3.12	5.35	2.41	4.81	2.40	5.57	4.71
MnO	0.57	0.42	0.42	0.32	0.25	0.39	0.25	0.30	0.28	0.25	0.24	0.32	0.37
NiO	0.09	0.05	0.08	0.09	0.11	0.13	0.14	0.13	0.12	0.11	0.11	0.12	0.11
MgO	47.55	47.29	50.04	50.22	52.37	52.56	53.62	52.44	52.32	52.37	54.25	51.20	52.02
CaO	0.01	0.01	0.03	0.02	b.d.	b.d.	b.d.	b.d.	0.02	b.d.	b.d.	b.d.	0.02
H_2_O^1^	3.13	2.92	3.40	3.11	2.07	1.98	2.13	2.34	1.70	2.07	2.27	2.11	1.97
Total	99.74	99.25	100.72	99.53	100.46	99.49	100.19	100.16	99.14	100.46	100.33	99.65	100.86
Si	1.978	1.997	1.979	1.993	3.981	3.979	3.991	3.981	3.988	3.981	4.053	4.005	4.013
Ti	0.377	0.414	0.336	0.388	0.269	0.299	0.251	0.171	0.393	0.269	0.198	0.241	0.301
Al	–	0.002	0.001	< 0.001	–	0.003	–	0.002	0.017	–	0.002	0.003	0.001
Cr	0.001	0.001	< 0.001	–	–	–	0.001	0.003	0.001	–	0.001	–	0.001
Fe^+2^	0.355	0.314	0.267	0.167	0.427	0.305	0.276	0.476	0.216	0.427	0.211	0.501	0.418
Mn	0.029	0.022	0.021	0.016	0.022	0.035	0.022	0.027	0.026	0.022	0.022	0.029	0.034
Ni	0.004	0.003	0.004	0.004	0.010	0.012	0.012	0.011	0.010	0.010	0.009	0.010	0.009
Mg	4.255	4.247	4.386	4.430	8.291	8.367	8.447	8.323	8.348	8.291	8.506	8.201	8.221
Ca	< 0.001	0.001	0.002	0.001	–	–	–	–	0.002	–	–	–	0.003
H	1.255	1.173	1.334	1.228	1.466	1.407	1.500	1.662	1.217	1.466	1.593	1.515	1.396
Mg#	91.6	92.6	93.8	95.9	94.8	96.0	96.5	94.2	97.1	94.8	97.2	93.8	94.7

Mg# = (Mg/(Mg + Fe + Ni + Mn))*100

Ti-ch is normalized to 7 cations and 18 charges and Ti-cl on 13 cations and 34 charges

*l* large grains (prograde first generation grains), *s* small grains (recrystallized), *p* prograde, *r* retrograde, *b.d.*  below detection limit

^1^H_2_O is recalculated from stoichiometry

**Table 6:** Major and minor element composition of accessory phases

Sample	FA	Ol26		14					Ol26	FA				FA	FA	Ol26	
Mineral	Grt	Grt		Ilm	Ilm	Ilm	Ilm		Prv	Bc	Bc	Bc	Bc	Serp	Cpx	Cpx	Cpx
wt%	incl in Cr–Mt	in vein		incl in Ti-ch	incl in Ti-ch	Large ilm	Large ilm										
SiO_2_	35.67	35.90		0.10	0.07	0.01	0.04		0.92	b.d.	b.d.	b.d.	b.d.	41.74	55.64	55.44	55.15
TiO_2_	0.56	0.06		58.45	58.13	56.33	57.93		53.87	b.d.	0.01	0.01	b.d.	b.d.	b.d.	b.d.	b.d.
Al_2_O_3_	0.16	0.09		b.d.	b.d.	0.02	0.01		b.d.	0.02	b.d.	0.01	b.d.	b.d.	0.01	b.d.	b.d.
Cr_2_O_3_	12.15	0.03		0.01	0.01	0.02	0.01		b.d.	0.04	0.02	0.04	0.02	0.07	0.01	b.d.	b.d.
Fe_2_O_3_	18.26	30.02		6.40	5.89	6.57	5.17		–	–	–	–	–	–	–	–	–
FeO	0.48	b.d.		9.16	9.95	18.64	13.23		0.67	4.03	4.40	5.92	6.07	2.29	0.50	0.45	0.36
MnO	0.29	b.d.		5.06	5.13	3.08	3.36		0.11	0.84	0.70	1.09	0.94	0.16	0.03	0.04	0.03
NiO	b.d.	b.d.		0.08	0.06	0.03	0.02		0.01	0.25	0.21	0.24	0.25	0.19	0.04	0.05	0.02
MgO	b.d.	0.04		21.50	20.84	16.17	19.87		0.31	64.80	64.67	62.71	62.70	42.80	18.08	17.79	18.04
CaO	33.08	34.59		0.01	0.01	0.02	0.01		41.31	0.08	0.07	0.07	0.08	0.06	26.33	26.52	26.65
H_2_O	–	–		-.	–	–	–		–	30.30	30.27	29.89	29.88	12.81	–	–	-.
Total	100.65	100.73		100.76	100.10	100.90	99.67		97.26	100.38	100.34	99.97	99.96	100.11	100.64	100.29	100.24
Si	2.981	2.998		0.002	0.002	< 0.001	0.001		0.021	–	–	–	–	1.953	2.000	2.001	1.993
Ti	0.035	0.004		0.946	0.950	0.944	0.957		0.932	–	<0.001	< 0.001	–	–	–	–	–
Al	0.016	0.009		–	–	0.001	<0.001		–	<0.001	–	<0.001	–	–	< 0.001	–	–
Cr	0.803	0.002		< 0.001	< 0.001	< 0.001	< 0.001		–	<0.001	< 0.001	< 0.001	< 0.001	0.003	< 0.001	–	–
Fe^2+^	0.034	–		0.165	0.181	0.348	0.243		–	0.033	0.036	0.050	0.051	0.090	0.015	0.014	0.011
Fe^3+^	1.148	1.887		0.104	0.096	0.110	0.085		0.013	–	–	–	–	–	–	–	–
Mn	0.021	–		0.092	0.094	0.058	0.063		0.002	0.007	0.006	0.009	0.008	0.007	0.001	0.001	0.001
Ni	–	–		0.001	0.001	0.001	<0.001		< 0.001	0.002	0.002	0.002	0.002	0.007	0.001	0.001	0.001
Mg	–	0.006		0.690	0.675	0.537	0.650		0.011	0.956	0.955	0.938	0.938	2.985	0.968	0.957	0.971
Ca	2.962	3.095		< 0.001	< 0.001	< 0.001	<0.001		1.018	0.001	0.001	0.001	0.001	0.003	1.014	1.025	1.032
H	–	–		–	–	–	–		–	2.000	2.000	2.000	2.000	4.000	–	–	–
Grossular	< 0.001	0.083	Hematite	0.052	0.048	0.055	0.043	Mg#	–	95.7	95.6	93.9	93.9	96.7	98.3	98.3	98.8
Spessartite	0.007	< 0.001	Ilmenite	0.164	0.181	0.348	0.243	Cat#	2.000	0.999	1.000	1.000	1.000	5.046	4.000	3.999	4.007
Andradite	0.577	0.913	Geikilite	0.689	0.675	0.538	0.651										
Uvarovite	0.404	0.001	Mn-Ilmenite	0.092	0.094	0.058	0.063										

*Grt* Garnet, *Ilm* Ilmenite, *Prv* Perovskite, *Bc* Brucite, *Serp* Lizardite/Chrysotile, *Cpx* clinopyroxene (diopside), *b.d.* below detection limit

Grt is normalized to 7 cations and 24 charges, Ilm on 2 cations and 6 charges, Prv on 3 oxygens, Bc on 2 oxygens, Serp on 9 oxygens and Cpx on 6 oxygens

Cat# = number of cations

Mg# = (Mg/(Mg + Fe + Ni + Mn))*100

– Not analysed or calculated

#### Spinel

**Fig. 9:**
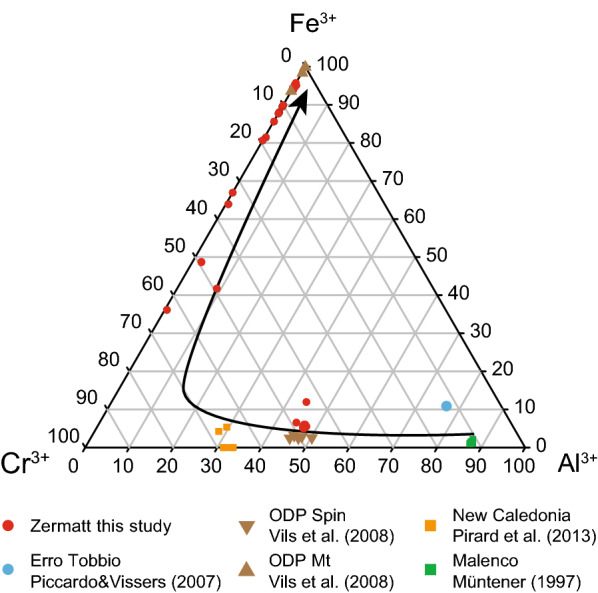
Spinel-ternary diagram showing the fertile mantle composition from Malenco (Müntener 1997) and Erro Tobbio (Piccardo and Vissers 2007) evolving to depleted harzburgites of Zermatt (this study), New Caledonia (Pirard et al. 2013) and ODP log 209 MAR from Vils et al. (2008) (Additional file 7: Table S3). Additionally, the expected retrograde overprint trend in meta-peridotites is indicated with the black arrow

The brown Cr-spinel cores in sample Ol28 (Table 1) lie on the compositional line between Cr and Al endmember at Cr# 0.5 typical for upper mantle compositions (Fig. 9). Cr-magnetite surrounding or replacing Cr-spinel (Fig. 3b, c) in FA and Ol28 shows elevated Cr_2_O_3_ contents of 25–46 wt% (Table 1). The recrystallized chromite rims magnetite veins and polygons contain only 1–4 wt% Cr_2_O_3_ (Table 1).

#### Olivine

**Fig. 10:**
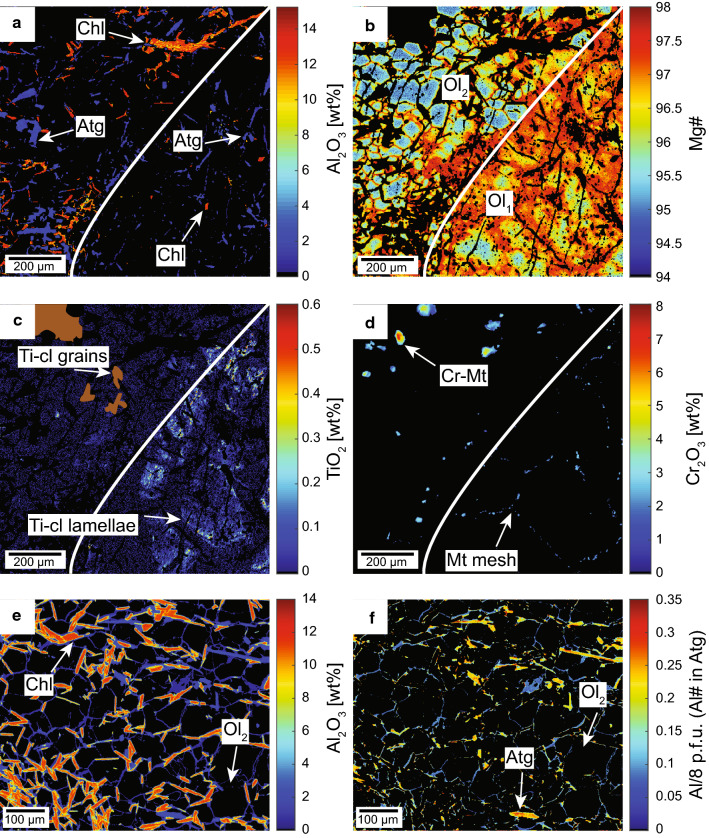
Quantitative major and minor element maps of a section in sample FA showing the two olivine generations, olivine_1_ (Ol_1_) on the right hand side of the white line and olivine_2_ (Ol_2_) to the left (**a**–**d**). The thin section image is given in Additional file 5: Figure S4. **e**–**f** Quantitative map of a different location within the same thin section FA. **a** Al_2_O_3_ content showing the distribution of chlorite and antigorite in both generations. **b** Mg# variation in both olivine generations, note the patchy zoning in olivine_1_ versus the apparently concentric zoning in olivine_2_. **c** Ti-clinohumite lamellae in olivine_1_ and discrete grains of Ti-clinohumite forming a mosaic texture with olivine_2_. **d** Relic magnetite mesh texture in olivine_1_ and relic Cr-rich magnetite in olivine_2_. **e** Al_2_O_3_ content of antigorite and chlorite forming a mosaic texture with “black” olivine_2_. **f** Al# indicating two generations of serpentine, high Al# antigorites forming a mosaic texture with chlorite and olivine, note the lower Al# towards their rims, and low Al# serpentine distributed around “black” olivine

Olivines from all studied samples including country rocks (AF2, MF, Ol28, FA), shear zone Ol1–Ol2 and olivine veins (Ol26, Ol46) show high Mg# 94–95 in the cores (Table 2, Figs. 8a, 10). The first generation olivine_1_ consisting of large olivine grains shows erratic patches of concentrically zoned Mg# 95–97 from core to rim and decreasing Ni with increasing Mg# (Figs. 8a, 10b). The second generation, olivine_2_, shows different populations among different samples with on average lower Mg# 94–95, but similar Ni contents compared with olivine_1_ (Fig. 8a). Trace element measurements of olivine_1_ returned mixed analyses due to the presence of numerous mineral inclusions. Successful analyses were only possible in clear, recrystallized olivine_2_ in samples FA, Ol1, Ol2 and Ol26. The trace element content relative to primitive mantle is very low in olivine_2_ (Fig. 8b), only B shows > 10 fold enrichment. Values for Li, Sc, Co, Ni and Zn are close to primitive mantle compositions, Mn is roughly two fold increased, whereas the remaining elements including the HREE are depleted (Fig. 8b). Al-contents range from 0.3 to 0.41 µg/g, whereas Cr-contents vary between 3.3 and 9.25 µg/g (Table 3).

#### Antigorite and chlorite

Our analyses were focussed on antigorite in textural equilibrium with olivine that formed close to peak metamorphic conditions or during early stages of retrogression. The serpentine generation occurring locally as late replacement veins (Fig. 7b, c) or halos (Fig. 7a) are volumetrically not important and not further considered here. Antigorite was recalculated to the polymorph m = 17 (Mellini et al. 1987). It shows core-to-rim zoning with elevated mass fractions of 2.2–2.8 wt% Al_2_O_3_ and 0.5–1.7 wt% Cr_2_O_3_ in the prograde cores and lower values at rims with little variation of Mg# 97–98 (Table 4). The rims developed coevally with late stage retrograde serpentine formation (Table 4). Decreasing Al-contents from core to rim are visible in the compositional map (Fig. 10f). Moreover, chlorite-bearing samples such as FA, Ol1-2, and Ol46 show generally higher Al_2_O_3_ mass fractions in antigorite than in chlorite-absent samples such as MF (Table 4). Chlorite contains 11–13 wt% Al_2_O_3_, between 0.7 and 2.6 wt% Cr_2_O_3_ and Mg# 95–96 in FA, Ol1-2, and Ol46 samples (Table 4, Fig. 10a, e).

#### Other minerals

A single Ti-chondrodite grain from the shear zone (Ol1) shows Mg# 96 and contains 8.7 wt% TiO_2_ (Table 5, Fig. 6c). Large grains from a Ti-chondrodite vein (sample 14) show Mg# 92–93 and TiO_2_ contents between 8.35 and 9.14 wt%. Small idioblastic grains in the Ti-chondrodite vein have Mg# 94 and TiO_2_ contents of 8 wt% (Table 5). Ti-clinohumites have Mg# 94–97 in Ol26, FA, AF2, Ol46 and MF and retrograde rims replacing Ti-chondrodites in the vein Mg# 95–96. TiO_2_ values vary between 2.1 and 4.9 wt% (Table 5). Both clinopyroxene (diopside) inclusions in olivine (sample FA) and the large grains in olivine veins (Ol26) show near endmember compositions with Mg# 98–99. Garnet in the country rock sample associated with Cr-magnetite and magnetite (FA) is characterised by a 60 mol% component of andradite and a 40 mol% component of uvarovite (Table 6). The garnet present in the olivine vein Ol26 consists of 91 mol% andradite and contains a small grossular component of 8 mol%. Ilmenite in sample 14 (Additional file 4: Figure S3) consists of 54–69 mol% geikilite and 16–35 mol% ilmenite endmembers and contain 4–6 mol% hematite and a 6–9 mol% Mn-ilmenite component. Perovskite grains in the olivine vein Ol26 show close to endmember compositions (Table 6). Retrograde brucite has Mg# 94–96 and is associated with lizardite/chrysotile of Mg# 97 and occasionally magnetite and calcite veins.

### Quantitative compositional mapping

An element distribution map was obtained from sample FA (Fig. 10a–d; see Additional file 5: Figure S4 for thin section image) that documents best the different olivine generations. The map is divided into two domains. In the right domain a large olivine_1_ grain contains inclusions of antigorite and chlorite and overgrows relic magnetite mesh textures (Fig. 10a, b). In the left domain, chlorite and antigorite form mosaic textures with idioblastic olivine_2_. Antigorite and chlorite contain 2–3 wt% and 10–12 wt% of Al_2_O_3_, respectively (Fig. 10a). The large olivine shows patchy zoning of randomly distributed cores with Mg# 95, surrounded by rim domains with Mg# 97. The recrystallized, polygonal olivine_2_ consists of cores with lower Mg# 95 and a small rim with Mg# 97 (Fig. 10b). In the mosaic domain, discrete polygonal Ti-clinohumite grains occur in textural equilibrium with olivine_2_ (Fig. 10c). On the contrary, Ti-clinohumite occurs as patches or lamellae surrounded by magnetite mesh polygons in the large olivine_1_ generation. A detailed map of the well-equilibrated olivine_2_ domain shows the mosaic texture between olivine, antigorite and chlorite (Fig. 10e, f). Magnetite in the mosaic domain displays decreasing Cr_2_O_3_ contents towards the rim, showing similar chemistry as the relic magnetite mesh polygons in the olivine_1_ domain (Fig. 10d). The Al# (Al a.p.f.u./8) indicates the growth of two distinct serpentine generations. The first shows high Al# > 0.3 while the second shows Al# < 0.1.

### H_2_O measurements

**Fig. 11:**
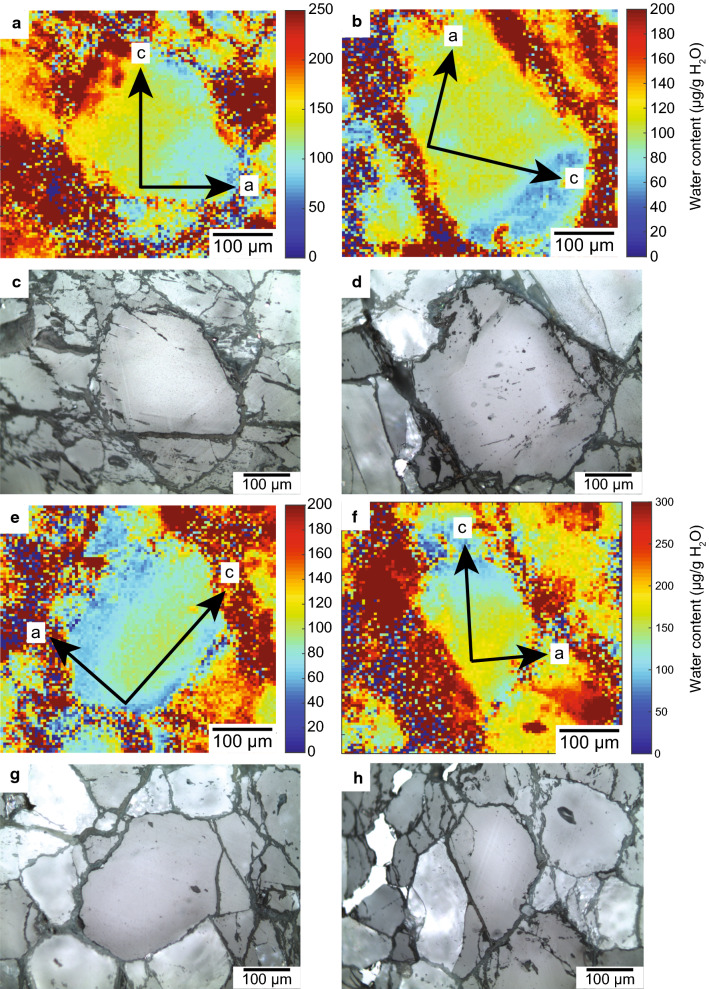
H_2_O concentration maps from the shear zone samples Ol1-2 in (µg/g) normalized to 1 cm thickness **a**, **b**, **e**, **f** and the corresponding reflected light image **c**, **d**, **g**, **h** respectively. Crystallographic axes are indicated with arrows and boxes. Reflected light images show the grain outlines and the dark rims are late retrograde olivine replacements by lizardite/chrysotile

The two generations of olivine were measured in free-standing thick sections with FTIR spectroscopy in order to determine H_2_O contents of metamorphic olivine. Both olivine generations contain 100–140 µg/g H_2_O, exclusively hosted in Si-vacancies where 4 H replace a Si cation (Kempf & Hermann, 2018). Kempf and Hermann (2018) have shown that this type of defect and the high water contents are diagnostic of olivine formed during the reaction antigorite + brucite = olivine + H_2_O. Additionally, a single H_2_O distribution map has been shown in that work, indicating that no significant H_2_O loss occurred after olivine formation. Here we mapped additional grains from the second generation olivine_2_ in sample Ol1. Water content maps of olivine_2_ display variation between 60 and 180 µg/g H_2_O (Fig. 11). Most of the grains show zoning unrelated to the principle crystallographic axis of olivine (Fig. 11a, b, f). However, in some grains zoning parallel to the a-axis can be observed (Fig. 11e, see also Kempf and Hermann (2018)). In this olivine (Fig. 11e) there is a gradual decrease from 110 µg/g in the core to 60 µg/g at the rim in a distribution that resembles diffusional loss. However, it is important to note that rim H_2_O mass fractions are still as high (60–100 µg/g) as in grains without any zoning pattern (Fig. 11b). This shows that no significant H_2_O has been lost during exhumation as suggested by Kempf and Hermann (2018). It is more difficult to quantify the H_2_O content of large and inclusion-rich olivine_1_ grains. Especially, tiny amounts of hydrous phases such as antigorite and chlorite can provide a large increase of absorbance in the relevant wavenumber region for the Si-vacancy point defect between 3500 and 3650 cm^−1^. In addition, tiny Ti-clinohumite inclusions present as grains or lamellae interfere in the main absorption region of 3500–3600 cm^−1^. This problem was addressed by only measuring the diagnostic 3613 cm^−1^ band in olivine_1_ for which there is no interference. From the spectra of clean olivine_2_ grains it is evident that the 3613 cm^−1^ band contributes 22% to the total absorbance. Thus, multiplying the integrated absorbance of this band in olivine_1_ with a conversion coefficient of 4.5, H_2_O contents of 100–110 µg/g are obtained, lying in the same range as olivine_2_ grains in the same sample FA 102 µg/g (Additional file 6: Table S4). This provides evidence that olivine_1_ and olivine_2_ formed at similar high-pressure conditions.

### Thermodynamic modelling

**Fig. 12:**
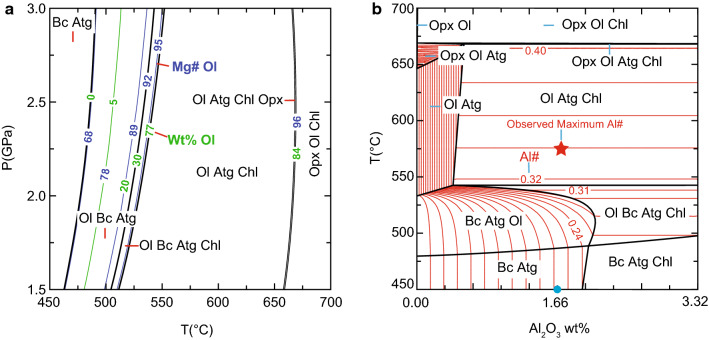
**a** P–T diagram calculated from the bulk composition extracted from the quantitative map (Fig. 10e, f). Mg# isopleths of olivine are in blue and wt%-isopleths of olivine in green. **b** T-X phase diagram calculated for a fixed pressure of 2.5 GPa varying only the Al_2_O_3_ content from Fig. 10f and assuming H_2_O saturation. The light blue dot marks the bulk Al_2_O_3_ content from the map extending the phase diagram to double the content 0.0–3.32 wt%. The red lines indicate the Al# numbers, Al/8 a.p.f.u

All phase diagrams were calculated for a local bulk composition extracted from the compositional map shown in Fig. 10 via XMapTools (see methods in Lanari and Engi 2017) using the Gibbs free energy minimizer Perple_X (Connolly 2009), with the thermodynamic dataset of Holland and Powell (1998) revised in 2002 (hp02ver.dat). The extracted bulk composition is MgO: 48.86 wt%, FeO: 3.25%, Al_2_O_3_: 1.66%, SiO_2_: 39.65% (Kempf and Hermann 2018). Olivine isopleths for Al#, Mg# and wt% were extracted with the Perple_X programme WERAMI. Solution models for olivine, orthopyroxene and brucite were taken from Holland and Powell (1998) and for chlorite from Holland et al. (1998). For antigorite, the aluminium tschermak exchange model of Padrón-Navarta et al. (2013) was used. Figure 12a shows the calculated P–T phase diagram for the local bulk map composition between 1.5 and 3 GPa and 450–700 °C. All assemblages are separated by steep boundaries. Olivine forms from brucite and antigorite first in a ~ 50 °C broad field where olivine, antigorite and brucite are stable. This olivine is expected to show increasing Mg# 68–92 with increasing temperature where between 0 and 30 wt% olivine is formed. At higher temperatures, chlorite is additionally formed in a narrow field of ~ 10 °C but 47 wt% olivine is formed in this field for the chosen bulk. At higher temperatures, brucite is exhausted and olivine, antigorite and chlorite coexist over a temperature interval larger than 100 °C. From ~ 660 °C orthopyroxene appears at first in a very narrow field of a couple of degrees where only 7 more wt% of olivine is formed.

Al# isopleths in antigorite were calculated according to Padrón-Navarta et al. (2013) and displayed in a T-X diagram (Fig. 12b) with variable Al_2_O_3_ content ranging from 0 to 3.32 wt% (double the original Al_2_O_3_ content) for 2.5 GPa between 450 and 700 °C (Fig. 12b). The other components of the bulk composition were kept constant. Isopleths of Al# exhibit two major trends. Steep curves indicate no or weak temperature dependence, whereas horizontal lines mean strong temperature dependence. More importantly, fields with horizontal lines contain chlorite while it is absent in fields with steep lines. The Al# in the Ol-Atg-Chl field varies between 0.32 at brucite-out to 0.40 before orthopyroxene-in reactions.

## Discussion

**Fig. 13:**
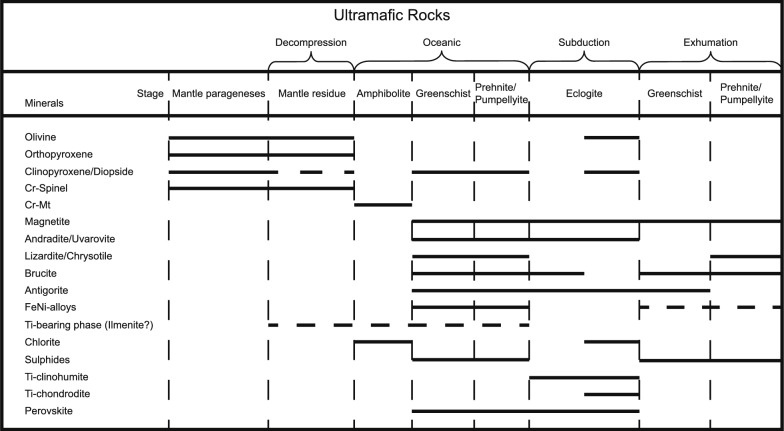
Parageneses present in the serpentinites at different metamorphic stages inferred from field observations, textures and mineral compositions. Solid lines indicate when a phase is stable whereas dashed lines are presumed partially present phases

Figure 13 summarizes the main metamorphic stages and associated mineral assemblages that can be deduced from a combination of field observations, thin section petrography and mineral analyses. In the following, we highlight the most relevant features of each stage and show that the serpentinites document a full orogenic cycle from rifting, through seafloor alteration, subduction and finally exhumation. Special emphasis is placed on the formation of brucite during oceanic serpentinization and the consumption of brucite to form metamorphic olivine during subduction. We present different ways of how such metamorphic olivine can be distinguished from mantle olivine. Then we determine the conditions and amounts of fluid that can be liberated by this reaction depending on whether a serpentinized lherzolite, harzburgite or dunite is subducted. Finally, the importance of this reaction for fluid liberation in subduction zones is discussed.

### Mantle and oceanic evolution

#### Mantle relics

**Fig. 14:**
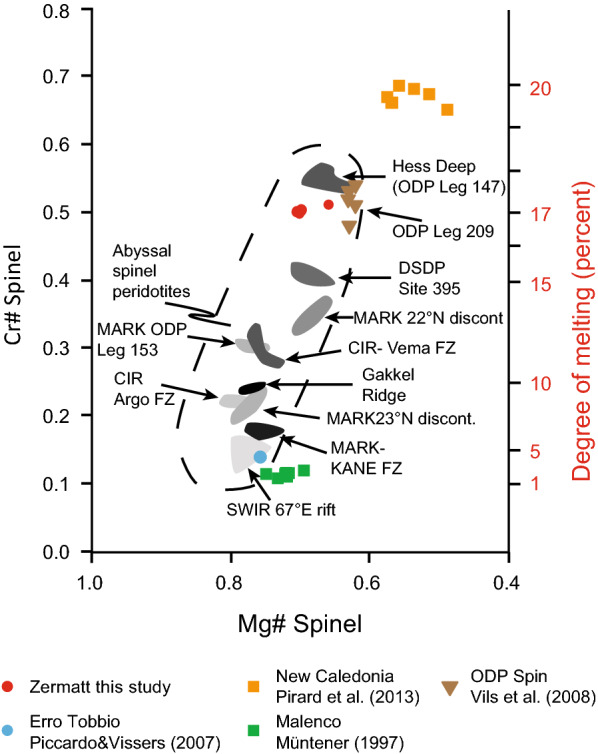
Representative spinel measurements from abyssal peridotites modified from Hellebrand et al. (2002); Vils et al. (2008), representative spinel core compositions of subducted serpentinites from Zermatt (this study) and Erro Tobbio (Piccardo and Vissers 2007), peridotites from Malenco (Müntener 1997) and New Caledonia (Pirard et al. 2013) (Additional file 7: Table S3)

The Cr-spinel cores (Fig. 3) are the only relics from the mantle protolith in the serpentinites (Fig. 14). Based on their composition, the Zermatt samples plot in the field of depleted abyssal peridotite. The high Cr# 0.5 indicates a significant melt depletion of ~ 17% according to Hellebrand et al. (2001) (Fig. 14, Additional file 7: Table S3). This result is consistent with the observation that there are only minor amounts of diopside in the serpentinites, indicating that most clinopyroxene was extracted in the Zermatt-Saas serpentinites during decompression melting (Hirschmann et al. 1998). This is supported with bulk rock data of the serpentinites that display a range in Al_2_O_3_ from 0.6 to 2.6 wt%, consistent with a depleted mantle origin (Li et al. 2004b). On the other hand, fertile peridotites are present in the Erro Tobbio, Voltri massive (Piccardo and Vissers 2007) and Malenco peridotites, which plot on the border of the abyssal peridotite array. These samples were described as subcontinental mantle (Müntener and Hermann 1996, 2001; Trommsdorff et al. 1993) showing little melt depletion and very low Cr# in Cr-spinel (Fig. 14). From these observations it is concluded, that the Zermatt-Saas serpentinites derive from a mantle section that might have been closer to an actual spreading centre than the exhumed subcontinental lithospheric mantle sections of Malenco and Erro Tobbio (Müntener and Hermann 1996; Piccardo et al. 1990; Rampone et al. 2005).

#### Mafic dykes

The ubiquitous meta-rodingites (Li et al. 2004a) as well as the less frequently observed Ti-chondrodite veins (meta-Ti-gabbro/basalt dykes) provide evidence of melt migration through the former mantle peridotite. Such meta-dykes are commonly interpreted to originate from basalts and Fe–Ti-Gabbro precursors (Gilio et al. 2019; Scambelluri and Rampone 1999; Shen et al. 2014, 2015), respectively, both intruding the local mantle during extension. In the least deformed section of the outcrop, meta-dykes form 90° angles to each other and cut the mantle tectonite foliation. This indicates that the outcropping rocks did not experience any shearing and deformation during the Alpine cycle as any deformation would lead to a lowering of the intersection angle and extensive boudinage of the meta-dykes.

#### Magnetite and brucite formation during serpentinization

The depleted peridotites were exhumed close to the ocean floor where serpentinization started. During ocean floor hydration the large relic Cr-spinels from the olivine-serpentinite partially recrystallized to Cr-magnetite rims (Fig. 3) including many sub-micron sized chlorites. Whether a nearly pure oceanic magnetite formed prior to the peak metamorphic rim cannot be inferred but also not excluded due to the presence of the magnetite mesh polygons. The magnetite mesh polygons, if present as inclusions of olivine_1_, are a direct proof for the hydration of mantle olivine. The high modal proportions of magnetite is explained by high temperatures during serpentinization > 325 °C (Klein et al. 2014). Therefore serpentinization might have taken place not directly at the seafloor, but at deeper levels in the oceanic lithosphere. This is in agreement with bulk δ^18^O values of serpentinites that are in the range of 1.5–4‰, indicative of high temperature serpentinization at 300–400 °C (Cartwright and Barnicoat 1999). Comprehensive serpentinite bulk rock analyses from the same area in Zermatt were presented by Li et al. (2004b) and were shown to be brucite normative because the Si/Mg ratios are lower than pure m = 17 antigorite (Bowen and Tuttle 1949; Coleman and Keith 1971; Hostetler et al. 1966). As FeO is the most important octahedral cation apart from MgO it has an important lever on the amount of brucite versus magnetite production during serpentinization. The more magnetite there is, the less brucite was formed (compare Table 7). Brucite formation occurred during seawater interaction with peridotites. This process has attracted a lot of attention in the literature because of its possible link to early life on Earth (Früh-Green et al. 2004). Studies from IODP samples show the presence of brucite in all studied localities (Bach et al. 2004; Beard et al. 2009; Früh-Green et al. 2004; Kodolányi et al. 2011). The reactions to form brucite, serpentine and magnetite have been thoroughly investigated (Beard et al. 2009; Beard and Frost 2016; Frost and Beard 2007; Klein et al. 2014; Moody 1976; O’Hanley 1996; Schwarzenbach et al. 2016). Accordingly, brucite forms dominantly via the reaction

1 $$\begin{aligned} 2({\text{Mg}},{\text{Fe}})_{2} {\text{SiO}}_{4} + 3{\text{H}}_{2} {\text{O}} = ({\text{Mg}},{\text{Fe}})_{3} {\text{Si}}_{2} {\text{O}}_{5}({\text{OH}})_{4} + ({\text{Mg}},{\text{Fe}})({\text{OH}})_{2} \;\pm  \;({\text{Fe}}_{3} {\text{O}}_{4} + {\text{H}}_{2}) \hfill \\ {\text{olivine}} + {\text{seawater}} = {{\text{lizardite}} \mathord{\left/ {\vphantom {{\text{lizardite}} {\text{chrysotile}}}} \right. \kern-0pt} {\text{chrysotile}}} + {\text{brucite}}      \;\pm\; ({\text{magnetite}} + {\text{hydrogen}}) \hfill \\ \end{aligned}$$

and in minor amounts from the reaction;

2 $$\begin{aligned} 17({\text{Mg}},{\text{Fe}})_{3} {\text{Si}}_{2} {\text{O}}_{5} ({\text{OH}})_{4} = ({\text{Mg}},{\text{Fe}})_{48} {\text{Si}}_{34} {\text{O}}_{85} ({\text{OH}})_{62} + 3({\text{Mg}},{\text{Fe}})({\text{OH}})_{2} \hfill \\ {{\text{lizardite}} \mathord{\left/ {\vphantom {{\text{lizardite}} {{\text{chrysotile}} = {\text{antigorite}} + {\text{brucite}}}}} \right. \kern-0pt} {{\text{chrysotile}} = {\text{antigorite}} + {\text{brucite}}}} \hfill \\ \end{aligned}$$

**Table 7:** Fluid production from the antigorite + brucite reaction, calculated vs measured

Bulk rock	Bc in wt %^1^	Bulk H_2_O^1^	T °C^1^	Bulk H_2_O^2^	T°C^2^	Olivine wt %^2^	Fluid wt %^2^	LOI	Mg# Ol^1,2,3^	Mg# Atg^1,2,3^	Mg# Bc^1,2,3^	Mg# Chl^1,2,3^	Mg# Cpx^1,2,3^	Density change^1/2^%^4^
Zermatt														
Lhz*	4.8	11.7	376	8.3	501	22.3	3.4	9.2	26^1^	93^1^	93^1^	98^1^	84^1^	2781/2922
									74^2^	97^2^	97^2^	99^2^	94^2^	5.1
									95^3^	93^3^			98^3^	
Lhz**	Talc present						No fluid							
Hzb*	6.75	13.2	359	8.4	512	30.8	4.8	11.3	21^1^	94^1^	92^1^			2716/2898
									80^2^	98^2^	98^2^			6.7
Hzb**	1.23	12.5	533	11.7	546	5.1	0.8							2649/2698
														1.8
Dun*	18.3	15.1	309	1.7	530	86.3	13.4	3.6	11^1^	91^1^	90^1^	99^1^		2696/3257
									89^2^	98^2^	98^2^			20.8
Dun**	12.1	14.6	529	5.5	546	55	11.5							2637/2954
														12
EPMA*	16.8	15.2	480	2.8	543	77.7	12.4	6.3	68^1^	97^1^	97^1^	99^1^		2636/3129
									95^2^	99^2^	99^2^	95^2^		18.7
									95^3^	97^3^				
EPMA**	14.6	15	533	4.1	551	66.8	8.9							2609/3023
														15.9
Primitive mantle, IODP														
PM*	4.3	11.3	400	8.4	501	19.5	2.9		31^1^	93^1^	93^1^	98^1^	85^1^	2795/2922
									74^2^	97^2^	97^2^	99^2^	94^2^	4.5
PM**	Talc present													
Hzb*	10.4	13.8	333	6.6	518	47	7.2		15^1^	91^1^	91^1^			2703/2989
									85^2^	98^2^	98^2^			10.6
Hzb**	4.8	13.2	529	9.8	544	20.6	3.4							2637/2772
														5.1
Dun*	13.3	14.2	287	4.8	518	60.8	9.4		8^1^	90^1^	89^1^			2711/3088
									86^2^	98^2^	98^2^			13.9
Dun**	6.7	13.6	529	8.7	530	29.1	4.9							2652/2815
														6.1

Ol-in calculated at 2 GPa, Bc-out at 2.5 GPa

Sources of bulk rock compositions: Zermatt bulk; for phase diagram computation from Li et al. (2004a, 2004b): Lhz = average bulk rock # 16–19 (meta-lherzolites), Hzb = average bulk rock #1–8 & 10–14 (meta-harzburgites), Dun = bulk rock sample lg 15 (meta-dunites)

EPMA bulk from the quantitative map (Fig. 10)

Primitive mantle (PM) from McDonough and Rudnick (1999) (Table 1)

IODP Hzb = average abyssal meta-harzburgites (n = 48), Dun = average abyssal meta-dunites (n = 20): From Deschamps et al. (2013) (Table 1)

1. LOI’s [wt% H_2_O] from Li et al. (2004a, 2004b). 2. As soon as talc is present brucite is absent

* All iron as FeO = FeO_tot_

** Bulk − FeO_tot_

^1^Calculated at Ol-in [wt% H_2_O]

^2^Calculated at Bc-out [wt% H_2_O]

^3^Natural olivines at Bc-out Measured with EPMA

^4^Density change in kg/m^3^ and in mass%

Note that after reaction (2), probably from early stages of subduction, the stable serpentine polymorph is antigorite. The Mg# of brucite is expected to be lower than that of the mantle olivine while the one for lizardite is expected to be higher. From the iron-rich brucite, secondary magnetite can form through oxidation which decreases the amount of brucite and increases its Mg# (Bach et al. 2006; Beard and Frost 2016). O’Hanley and Dyar (1993) have shown that lizardite can incorporate more Fe^3+^ than antigorite. Therefore, at the transition of lizardite to antigorite, additional magnetite might have formed. Redistribution of SiO_2_ during serpentinization from sediments and seawater will consume brucite. IODP samples mostly originate from the uppermost hundreds of meters in an oceanic crust and have a limited access to the deeper levels where fluids and sediments have a decreasing potential to influence serpentinization. Modal brucite formation during serpentinization is dependent on the degree of melt depletion of the primitive mantle, showing increasing modal olivine content (and thus decreasing Si/Mg) with increasing depletion, mainly due to pyroxene extraction. The extent of brucite formation in the variably depleted oceanic mantle is then linked to the degree of serpentinization of the oceanic crust at depth. The degree of serpentinization is important since olivine is the first phase to serpentinize while orthopyroxene is more stable and thus relatively more brucite is formed at depth (Schwarzenbach et al. 2016).

### Prograde metamorphic olivine growth

#### Phase analysis

There are not many metamorphic reactions that allow the construction of detailed prograde P–T paths in serpentinites. Reaction 2 roughly coincides with the transition from sub-greenschist to greenschist facies (Evans 2004; Trommsdorff and Evans 1974). Then, the antigorite + brucite reaction provides an important marker to distinguish between greenschist/blueschist facies conditions (olivine free) and epidote–amphibolite/eclogite facies conditions where metamorphic olivine is present. The MgO-SiO_2_-H_2_O is the simplest chemical system where forsterite is formed along an univariant reaction:

3 $${\text{antigorite}} + {\text{brucite}} = {\text{olivine}} + {\text{H}}_{2} {\text{O}}$$

With the additional component Al_2_O_3_ present in antigorite as Al-Tschermak a new phase needs to form on the product side of the equation which is chlorite:

4 $${\text{antigorite}} + {\text{brucite}} = {\text{olivine}} + {\text{chlorite}} + {\text{H}}_{2} {\text{O}}$$

Adding ferrous iron to the system transforms univariant lines into divariant fields. Based on a reactive bulk composition extracted from compositional maps (Fig. 10e, f), olivine formation is predicted to occur within ~ 50 °C where antigorite, brucite and olivine are stable and additionally in a narrow ~ 10 °C field towards higher temperatures where chlorite is formed (Fig. 12a). The erratic distribution of lower Mg# patches (Fig. 10b) in olivine_1_ is explained by Fe–Mg-mixing properties of the olivine solid solution where Fe-rich olivine becomes progressively more Mg-rich with increasing temperature seen in the blue Mg# isopleths in Fig. 12a. For metamorphic olivine formed during serpentinite subduction this behaviour was studied by Kunugiza (1982) in the Sanbagawa metamorphic belt and is equivalent for the formation of the olivine_1_ generation.

#### Olivine zoning

Olivine_1_ growth started forming more iron-rich patches with Mg# 95 first, becoming increasingly MgO-enriched towards rims (Mg# 97) (Figs. 8a, 10b). The distribution of the lower Mg# 95 nuclei may be governed by the initial distribution of FeO in the reactant minerals antigorite and brucite. The overall very high Mg# is related to the high amount of magnetite in the sample that sequesters a significant proportion of the Fe in the bulk rock. The lower average Mg# 94–95 of olivine_2_ (Fig. 10b), that clearly originates from recrystallization of olivine_1_, is not in agreement with the expected Mg# increase at higher temperatures (Fig. 12a). A possible explanation is the consumption of the magnetite mesh polygons as observed (Figs. 5b–d). This requires either Fe^3+^ to be reduced to Fe^2+^ in order to be incorporated by olivine or while ferrous iron is incorporated in olivine ferric iron is hosted in coexisting minerals. There are several possibilities how this could be achieved. (1) The simplest explanation is that magnetite reacted with relic alloys to form a FeO component that was incorporated into olivine. The presence of andradite-uvarovite garnet with magnetite (Mt) and occasionally with diopside and or chlorite in sample FA (Fig. 4g) and olivine veins Ol26 (Fig. 6f, g) suggests the former presence of metal alloys such as taenite-awaruite at the ocean floor stage (Frost 1985). The absence of FeNi-alloys in the prograde assemblage suggests their participation in the olivine forming reactions. (2) A second hypothesis is that magnetite reacted with sulphides releasing Fe^2+^, while the sulphides were oxidised (Merkulova et al. 2017). The produced SO_2_ would then be able to leave the system with the water-rich fluid. However, a recent study has shown, that the fO_2_ for the antigorite + brucite to olivine reaction is well below QFM and that SO_2_ is only present in trace amounts in the fluid at the corresponding P–T conditions (Piccoli et al. 2019). (3) While the FeO component of magnetite is incorporated into olivine, some MgO of olivine is incorporated into magnetite and the ferric iron (or Cr in the case of Cr-magnetite) might be accommodated by existing or newly formed phases. For example, the high Cr-contents in both peak metamorphic antigorite and chlorite (Table 4) can be explained by the consumption of magnetite and Cr-magnetite that contain 4 wt% and 46 wt% Cr_2_O_3_, respectively. Another possibility is that new andradite garnet is formed hosting the ferric iron liberated by magnetite breakdown. In any case, magnetite does not seem to be fully inert during the olivine forming reaction. Also, two possibilities are suggested for sulphide participation due to the observed scarcity of sulphides in the olivine_2_ domain. (1) They partially re-equilibrated where their iron component (ferrous) partitioned into olivine while their Ni-content passively increased in sulphides, thereby decreasing the volume of sulphides present. (2) At the low oxygen fugacity, sulphur might be lost as H_2_S (Piccoli et al. 2019) decreasing the amount of sulphides and making Ni and Fe available for olivine. Considering thermodynamic equilibrium, it is unlikely that the sulphides remained inert during the olivine forming reaction.

#### Al-in-antigorite and olivine

The Al# isopleths for antigorite show the temperature dependence of the tschermak exchange in antigorite once its Al content is buffered by an Al-rich phase (Padrón-Navarta et al. 2013). This is achieved when chlorite becomes stable. The maximum Al# from the map is 0.32 (Fig. 10f), in line with the modelled maximum values from Fig. 12b. This indicates a minimum temperature of 520 °C at a pressure of 1.5 GPa and a maximum temperature of 560 °C at a pressure of 3.0 GPa. Moreover, Padrón-Navarta et al. (2013) discussed the influence of elevated Cr_2_O_3_ mass fractions in natural antigorite on the Tschermak substitution thermometer which was calibrated on 0.21–0.34 a.p.f.u. Cr contents. Because, peak antigorite (Table 4) in Zermatt attains up to 1.03 a.p.f.u. Cr producing (Al + Cr)# 0.34–0.38. This suggests ~ 30–60 °C higher peak temperatures than calculated here. Trace element measurements showed that metamorphic olivine contains 0.3–0.41 µg/g Al. Applying the pure Al-in-olivine thermometer (without Cr) returns temperatures between 575–590 ± 20 °C (De Hoog et al. 2010), in excellent agreement with the other determinations. Therefore, the maximum temperature of peak metamorphism is closer to the antigorite + brucite reaction rather than the antigorite-out reaction as suggested in other works (Bucher and Grapes 2009; Zanoni et al. 2016) and also supported by the absence of orthopyroxene in the area. On the other hand, peak temperatures as low as 500 °C suggested by Weber and Bucher (2015) can also be excluded due to the observed mineral assemblage.

A word of caution is required for the application of Al-in-antigorite as a thermometer. Figure 10f shows the second serpentine generation growing around polygonal olivine grains. Their very low Al# clearly separates them from peak antigorite whose Al content is diluted at rims. The dilution of the Al# indicates partial re-equilibration with late serpentine and is likely experienced when measuring antigorite in spot analyses mode with EPMA i.e., instead of obtaining Al# 0.32 as in Fig. 10f a lower value of Al# 0.29–0.31 is obtained in all samples (Table 4). Peak temperatures are thus prone to being underestimated. On the other hand, the lower values are also generated by overlap measurements over the zoned antigorite blades owing to the necessary beam diameter spreading during EPMA measurements. This is commonly done to avoid beam damage in the measured serpentine grains. Furthermore, this clearly shows the value of the mapping technique even though high currents are used, calibration with spot analyses guarantees the link between X-ray intensity and composition.

#### Pressure constraints

**Fig. 15:**
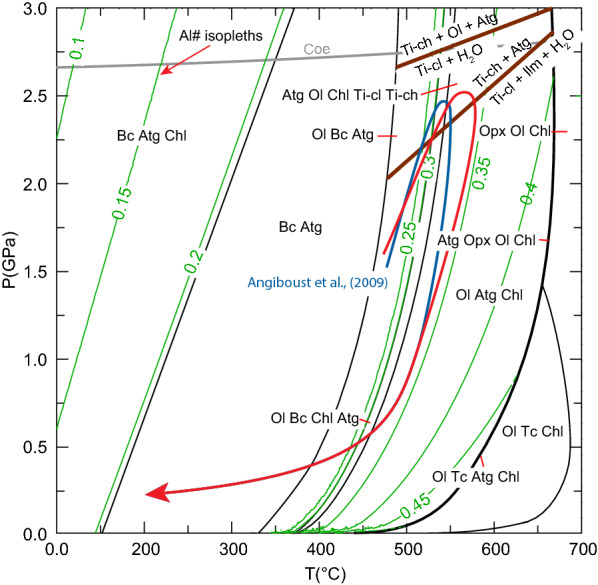
P–T diagram of the serpentinites: Stable assemblages are indicated and bordered by black boundary fields. Al#-isopleths of antigorite in green indicate the temperature dependence and suggest the values expected in natural samples using the respective bulk rock composition. Al# > 0.3 are present as soon as chlorite becomes stable. The brown lines represent reactions involving Ti-clinohumite and Ti-chondrodite from Shen et al. (2015). The suggested prograde P–T path from this study is indicated with the red line in comparison with the P-T evolution of Angiboust et al. (2009) in blue

Pressure constraints can be deduced from the Ti-clinohumite/Ti-chondrodite equilibria (Shen et al. (2015) in the shear zone and in the Ti-Chondrodite vein (Fig. 15). Ti-chondrodite can be formed through the reaction:

5 $${\text{Ti-clinohumite}} + {\text{ilmenite}} + {\text{H}}_{2} {\text{O}} = {\text{Ti-chondrodite}} + {\text{antigorite}}$$

This indicates that once the Ti content in Ti-clinohumite is buffered, Ti-chondrodite forms at the expense of Ti-clinohumite between 480 °C and 2.0 GPa and 660 °C and 2.8 GPa. However, at higher pressures, Ti-clinohumite disappears along

6 $${\text{Ti-clinohumite}} + {\text{H}}_{2} {\text{O}} = {\text{Ti-chondrodite}} + {\text{olivine}} + {\text{antigorite}}$$

between 490 °C and 2.6 GPa and 670 °C and 3.0 GPa. Ti-clinohumite is present as minute lamellae dispersed within the large olivine_1_ as well as polygonal grains within the recrystallized olivine_2_ (Fig. 10c). Ti-chondrodite coexists with Ti-clinohumite and is abundant in the Ti–rich veins, where precursor ilmenite must have been present. Most grains present in the shear zone, shear bands and olivine veins consist of Ti-clinohumite. A single Ti-chondrodite grain was observed in the shear zone (sample Ol1-2). This grain is strongly retrogressed to Ti-poor magnetite and Ti-clinohumite but no ilmenite is present, suggesting a possible Ti-chondrodite formation via reaction (6). However, the shear zone is juxtaposed to a metarodingite generally enriched in Ti. The key observation is that olivine, Ti-chondrodite and Ti-clinohumite coexist, which provides a good constraint for pressure when coupled with the tschermak model for antigorite and Al-in-olivine thermometry. For the peak temperature of 570–590 °C, the pressure range where Ti-chondrodite and Ti-clinohumite coexist is between 2.3 and 2.9 GPa.

Ti-chondrodite has been described in the Zermatt-Saas serpentinites to the south in the Valtournanche valley, Italy. A possible UHP origin of these serpentinites was suggested by Luoni et al. (2018). However, Ti-chondrodite is generally associated to Ti-rich lithologies (see their Appendix) and coexists with Ti-clinohumite. Ti-chondrodite also occurs in veins in serpentinites close to the UHP unit of Lago di Cignana, but also there it derives from former Ti-rich mafic dykes (Gilio et al. 2019).

### Comparison with other rocks

#### Peak conditions

Published peak temperatures and pressures of 550–600 °C and 2.5–3.0 GPa in nearby eclogites (Bucher et al. 2005), between 600 and 610 °C and 2.5 GPa in meta-gabbros (Bucher and Grapes 2009) are in good agreement with our thermobarometric data. The lower end of these pressure estimates is preferred due to the absence of coesite (Coe) in the meta-mafic and meta-sedimentary rocks of the ophiolite sequence (see Fig. 15). We derive from the combined evidence that olivine formation occurred close to peak P–T conditions on the prograde path through the involvement of brucite at temperatures of 550–600 °C and pressures > 2.0 GPa but < 2.7 GPa (Fig. 15). A comprehensive overview of P–T estimates for the whole Zermatt-Saas zone eclogites is provided by Angiboust et al. (2009). Their estimates of 540 ± 20 °C and 2.3 ± 0.1 GPa are in excellent agreement with our peak metamorphic conditions. Similar estimates, 500 ± 50 °C and 2.2 ± 0.2 GPa (de Meyer et al. 2014), were reported from the metasedimentary cover of the Zermatt-Saas ophiolite at Triftji (Fig. 1) that is surrounded by the olivine-bearing serpentinites described in this study.

Bucher et al. (2005) suggested that the eclogites at Pfulwe formed through external fluids from serpentine breakdown. Bucher and Grapes (2009) showed that over 90% of the eclogite facies peak parageneses in the Allalingabbro formed due to large amounts of external fluids at the peak P–T as did Li et al. (2008) in the eclogites facies metarodingites throughout the Zermatt-Saas serpentinites. As shown here antigorite breakdown conditions were never attained. The determined P–T of eclogite transformation coincides well with the peak P–T estimates of the antigorite + brucite reaction presented here. Thus, the good overall agreement between P–T estimates from ultramafic, mafic and sedimentary rocks shows that all rocks equilibrated close to peak metamorphic conditions possibly triggered by the large amount of fluid released by the antigorite + brucite reaction.

#### Retrograde path

The presence of fluids during exhumation is documented by talc-carbonate veins since they crosscut the prograde olivine-serpentinite (Fig. 7a). Additionally, antigorite veins pseudomorphically replace metamorphic olivine which indicates the presence of Si-bearing fluids due to the absence of brucite (Fig. 7b, c). Both assemblages of these veins could have formed anywhere on the retrograde path after olivine formation. An important constraint from the serpentinites is that the exhumation path does not cross the talc + olivine field meaning that a low-pressure antigorite-breakdown has not occurred (Fig. 15). Greenschist facies overprint at 0.4–0.6 GPa and 400–450 °C in the meta-mafic rocks was described in several studies (Bearth 1967; Bucher et al. 2005; Cartwright and Barnicoat 2002; de Meyer et al. 2014; Reinecke 1991; Rubatto et al. 1998). The nearly isothermal decompression (fast exhumation path) in the area suggested by many studies (Agard et al. 2009; Cartwright and Barnicoat 2002; de Meyer et al. 2014; Reinecke 1991; Rubatto et al. 1998) is in line with our observations.

### Metamorphic olivine derived from the antigorite + brucite reaction

#### Textures, major, minor and trace elements

Recognition of metamorphic olivine formed from antigorite + brucite is challenging since it is variably abundant throughout serpentinites in the field and is often strongly overprinted. For example, most serpentinites involved in subduction, exhumation and obduction are strongly sheared. Pre-alpine textures are thus often smeared out and retrograde overprint partially re-serpentinizes metamorphic olivine with and without brucite formation (Fig. 7). Recognizing metamorphic olivine is important as it provides a documentation of fluid-producing reactions during subduction. The following set of tools can be used to distinguish metamorphic olivine of the antigorite + brucite reaction from mantle olivine.

**Fig. 16:**
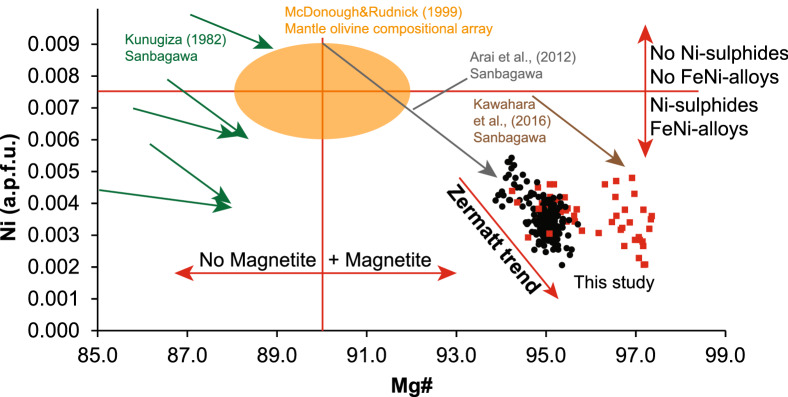
Ni vs Mg# trends in prograde metamorphic olivine in Zermatt samples, red symbols olivine_1_ and black symbols olivine_2_ (data from Fig. 8a and Additional file 3: Table S2), compared with the Sanbagawa metamorphic belt in Japan (Arai et al. 2012; Kawahara et al. 2016; Kunugiza 1982)(core to rim trends indicated with arrows). The orange field indicates the mantle array of Ni (a.p.f.u.) vs Mg# (McDonough and Rudnick 1999). Red lines show the controls of magnetite, FeNi-alloys and sulphide presence on olivine compositions that derive from the antigorite + brucite reaction

The olivine_1_ generation clearly overgrows magnetite polygons (Figs. 5b, 10d) typically representing the mesh texture and magnetite veins formed during ocean floor hydration of mantle olivine (O’Hanley 1996; Wicks and Whittaker 1977). The presence of Ti-clinohumite lamellae (clearly a metamorphic mineral) in olivine_1_, in the core of the magnetite polygons, (Fig. 10c) indicates metamorphic growth of olivine. From the major element composition, the very high Mg# 95–97 (Fig. 10b) is explained by the coexistence with Cr-magnetite and magnetite and deviates significantly from the compositional array of upper mantle olivine (Mg# 88–92, see Fig. 16). NiO in metamorphic olivine (0.11–0.27 wt%) is generally lower than in mantle olivine (0.4 wt%; reported in McDonough and Rudnick (1999) whereas MnO shows elevated concentrations (0.16–0.36 wt%,) compared to mantle olivine (0.14 wt%). Various studies on metamorphic olivine in the Sanbagawa belt in Japan found comparable core-rim trends (Arai et al. 2012; Kawahara et al. 2016; Kunugiza 1982) to what is observed in our study (Fig. 16). The lower NiO content is due to the presence of Ni-sulphides (pentlandite e.g. Piccoli et al. 2019), the large amount of magnetite and the former presence of FeNi-alloys (taenite-awaruite). The elevated MnO in olivine points to a co-existence with antigorite that has MnO contents below 0.05 wt%. A typical trace element marker for metamorphic olivine from dehydration reactions in oceanic lithosphere is B, since it is enriched in seawater (Scambelluri et al. 2001, 2004, Seyfried and Dibble 1984; Tenthorey and Hermann 2004; Thompson and Melson 1970; Vils 2009; Vils et al. 2011, 2008). However, to our knowledge, there is currently no comprehensive study of B systematics in pristine mantle olivine except for a couple of analyses on Kimberlites (Kent and Rossman 2002). Therefore, all data were normalized to primitive mantle compositions from McDonough and Sun (1995). The greater than tenfold enrichment of B relative to primitive mantle values (8b and Table 3) is a further clear indicator for a seawater signature (Kodolányi et al. 2011). Additionally, most trace element contents are lower than primitive mantle olivine values. Another good example is Al that has been used as a thermometer in peridotites (De Hoog et al. 2010). Equilibration temperatures of 800–1400 °C result in highly variable Al contents of 10–800 µg/g Al. The lower formation temperatures of olivine from the antigorite + brucite reaction leads to distinctly lower Al contents of 0.3–0.41 µg/g. Therefore, textural and compositional data can help for the identification of olivine that grew from the antigorite + brucite reaction.

#### Water in olivine

Hydrogen incorporated into Si-vacancies conveniently measured with FTIR spectroscopy is another very strong indicator for the presence of brucite during olivine formation. The antigorite + brucite reaction (4) produces H_2_O and thus favours a high water activity in the free fluid phase. At the same time the assemblage in the divariant field antigorite-brucite-chlorite-olivine imposes a low SiO_2_ activity such that Si-vacancies form in the product olivine (Kempf and Hermann 2018). The mapped H_2_O distribution in randomly oriented olivine grains displays no systematic relationships with the crystallographic axis (Fig. 11). There is no sign of a normal tracer diffusional loss of H_2_O. Even if a few grains display concentric zoning, H_2_O mass fractions never go to zero at rims and can thus be interpreted as growth zoning (Kempf and Hermann 2018). Water contents in the olivine_2_ (sample Ol1-2, shear zone) generation presented in the maps are well above 100 µg/g in agreement with the values provided by Kempf and Hermann (2018). The water contents of the inclusion-rich, large olivine_1_ generation, are within uncertainty identical with olivine_2_. The core-rim contents can vary by > 50%, generally decreasing towards the rims. FTIR spectra of olivine_1_ also display absorption at 3370–3400 cm^−1^, which is typical for Ti-clinohumite lamellae (Shen et al. 2014) and thus a metamorphic origin of the olivine.

The combined evidence from textures, composition and FTIR spectroscopy demonstrates that all olivine in the serpentinite has grown from the antigorite + brucite reaction on the prograde path. This contradicts previous studies that suggested that such olivine represents recrystallized mantle olivine (Li et al. 2004b; Rahn and Rahn 1998).

#### Metamorphic olivine as a proxy for brucite distribution in the field

It is notoriously difficult in lower grade serpentinites to detect brucite due to its small grain size and the tendency to intergrow with serpentine minerals. As we have established, that all the olivine in the mapped outcrop derives from the antigorite + brucite reaction, we can now use the distribution of olivine to infer the former distribution of brucite. In the least deformed part of the serpentinite there is a pronounced stripe pattern at the 10–20-cm scale consisting of olivine-bearing dark versus the olivine-absent green serpentinites, providing evidence for a heterogeneous brucite distribution at the mesoscale (Fig. 3a). In other undeformed areas, olivine occurs in patches that are distributed more evenly throughout the rock (Fig. 5a). A significant proportion of the outcrop does not contain olivine at all, indicating that these parts may derive from a brucite-free protolith. As discussed above, the formation of brucite during ocean floor alteration depends on many factors including variation in bulk Si/Mg as well as changing formation conditions. The eclogite-facies olivine bearing serpentinite might thus still document major alteration processes that occurred at the ocean floor. More detailed studies, especially including oxygen isotopes, are needed to fully exploit this information.

#### Olivine as marker for fluid pathways in subducted serpentinites

The antigorite + brucite reaction produces 30 wt% of olivine_1_ and a total of 77 wt% olivine_1_ and olivine_2_ according to the calculated model shown in Fig. 12a at reactions (3, 4), leading to a significant change in density and volume of the involved serpentinite for this olivine-rich local bulk rock composition (Table 7). This allows fluid to be pooled first at grain boundaries (Fig. 5d) in agreement with the model of Plümper et al. (2017). The fluid produced is subsequently flowing along grain boundaries and thought to form small fluid conduits visible as shear bands in the field gathering into broader shear zones (Fig. 6a, b, e) and olivine veins (Fig. 6d) as also supported by the focused fluid flow model of Plümper et al. (2017). Therefore, in the more deformed parts of the outcrop, the distribution of metamorphic olivine can be used to trace the pathways of the escaping fluid from reactions (3, 4). Olivine veins are often aligned en-echelon along the shear zones, providing evidence for synkinematic precipitation of olivine in veins with movements along the shear zones. The exact link between this fluid release and the mylonite formation is not yet clear. Nevertheless, the significant fluid release and associated volume decrease (Table 7) in shear zones would be large enough to potentially trigger deep earthquakes. The fluid passes also through metarodingites where it dissolves diopside, andradite and perovskite and precipitates them in olivine veins (Fig. 6d). How far certain elements are able to travel within this vein-shear zone system requires also further investigation.

#### Implications of hydrous olivine on slab rheology and the deep water cycle

Olivine-rich rocks such as olivine veins, shear zones and olivine-rich meta-harzburgites produced by the antigorite + brucite reaction are more competent than the neighbouring serpentinites. However, this relationship might change after the breakdown of chlorite and antigorite during further subduction. The olivine derived from the antigorite + brucite reaction contains up to 10 times more H_2_O than what is expected at the antigorite-out reaction and about 4 times more water than at the chlorite-out reaction (Padrón-Navarta and Hermann 2017). H replacing Si has a strong influence on the rheology of olivine-dominated rocks (Faul et al. 2016). Therefore olivine-rich shear zones that derive from the antigorite + brucite reaction might be weaker than the later produced olivine from antigorite- and chlorite-breakdown and play an important role for strain localisation within the slab. This hypothesis is based on the assumption that there is no diffusional equilibration of H in Si-vacancies in olivine with further subduction. A recent experimental study on the Zermatt olivines (sample Ol1-2) from this outcrop by Jollands et al. (2019) has shown that at atmospheric pressures the Si-vacancies are dehydrogenated within hours at elevated temperatures. However, the study also showed that dehydrogenation in 1 atm experiments is not easily extrapolated to natural conditions and that H loss or gain is governed by a complex interplay of inter-site reactions and diffusion.

### Modelled vs. observed mineral compositions: The temperature interval of fluid release

A close inspection of the phase diagram results (Fig. 12a) suggests that the first olivine nucleus should show Mg# as low as 68 at the olivine-in reaction, which is significantly lower than the observed Mg# 95 crystallization centres of olivine_1_ (compare Fig. 10b with Fig. 12a). In the first olivine_1_ bearing field 30 wt % olivine with Mg# 68–92 is expected to produce a volume large enough to be detected in thin section. At the brucite-out boundary, however, Mg# 94–95 is in agreement with natural observations in olivine_1_ and olivine_2_ (Table 7). Overstepping of the olivine-in reaction by ~ 50 °C involving two hydrous phases seems unlikely. Moreover, Fe–Mg diffusive equilibration should not occur for this temperature range at the given time frame (Chakraborty 2010) and thus cannot have obliterated a Fe-rich olivine. Antigorite and chlorite show Mg# 99 (Table 7) when modelled at brucite-out and the natural examples show Mg# 97 and Mg# 95 respectively. Brucite and antigorite show calculated Mg# 97 at olivine-in compared with Mg# 68 for olivine_1_. This discrepancy shows that calculated Mg# in antigorite, chlorite and possibly brucite are too high forcing iron to partition into olivine. This has the consequence that metamorphic olivine from the antigorite + brucite reaction starts forming at too low temperatures and shows an exaggerated fayalite component. Consequently, the divariant field where olivine and brucite coexist becomes too broad in the phase diagram. This effect is more accentuated when serpentinites with a reactive bulk of Mg# 90 is used instead of the local bulk map composition (Mg# = 96) where most bulk iron is stored in magnetite. For example, in the Zermatt-lherzolites, harzburgites and dunites, the divariant field between olivine-in and antigorite + brucite would expand from 376 to 501 °C (∆T = 125 °C), 359–512 °C (∆T = 153 °C) and 309–530 °C (∆T = 221 °C) with olivine Mg# of 26–74, 21–80 and 11–89, respectively. Similar large ranges are observed for primitive mantle and IODP samples from meta-harzburgite and meta-dunite respectively (Table 7). On the contrary, even where magnetite has not extracted ferrous iron from the reactive phases such low Mg# in olivine are not attained in nature (Fig. 16). This suggests that the solution models for Mg–Fe exchange in brucite-antigorite-chlorite-olivine equilibria are insufficiently constrained and erroneously force ferrous iron partitioning into olivine relative to the other phases. Divariant fields must be significantly smaller (Trommsdorff and Evans 1972) since in natural examples Mg# < 85 are hardly attained around the world (Fig. 16) suggesting a width of the divariant field of ~ 20 °C (Fig. 12a). Even the lowest Mg# 80 olivines (Scambelluri et al. 1991) would extent the divariant field to only ~ 30 °C and little olivine could be produced between Mg# 80–85. This would reduce the extension of the divariant field, where olivine and brucite coexist, to less than 30 °C, and thus limit also the temperature interval, where fluid is released.

### Bulk rock fluid release from the antigorite + brucite reaction

The maximum and minimum weight percentage of fluid released from the antigorite + brucite reaction in the Zermatt-Saas serpentinites was forward modelled using Perple_X’s subroutine Meemum in the FMASH and CFMASH system for dunites, harzburgites and lherzolites, respectively. The results of the modelling are summarized in Table 7. As discussed above, the amount of magnetite influences how much brucite is formed during serpentinization, which in turn influences the amount of fluid that can be liberated at the antigorite + brucite reaction. All Fe is considered in the model as FeO resulting in a maximum estimate for the bulk fluid release. For the minimum estimate we imposed that all FeO was extracted from the system producing only olivine Mg# 100 at the termination of the reaction. A meta-dunite (MD) (Lg15), average meta-lherzolites (ML) and meta-harzburgite (MH) from Li et al. (2004b) and the local bulk map composition (this study) (Table 7) were used for the modelling. For FeO = FeO_tot_, average MD, MH and ML water contents stored in solids prior to olivine growth is 15.1 wt%, 13.2 wt% and 11.7 wt% H_2_O. The calculated bulk H_2_O content in solids after olivine formation is 1.7 wt% (MD), 8.4 wt% (MH) and 8.3 wt% (ML) H_2_O. This results in a highly variable amount of fluid that is produced during the antigorite + brucite reaction ranging from 13.4 wt% (MD) to 4.8 wt% (MH) to 3.4 wt% H_2_O (ML). This amount is significantly reduced if FeO_tot_ is present as magnetite to 11.5 wt%, 0.8 wt % and 0.0 wt% H_2_O, respectively. The local bulk composition from the map shows a modelled bulk H_2_O content before and after olivine formation of 15.2 wt% and 2.8 wt% H_2_O suggesting a maximum loss of 12.4 wt% H_2_O. These calculations demonstrate that highly variable and especially for highly depleted serpentinites and dunites large quantities of H_2_O can be liberated at the antigorite + brucite reaction.

The calculated H_2_O values can be compared with the measured LOI’s from Li et al. (2004b) assuming no carbonate contamination. The LOI are 3.6 wt% (MD), 11.3 wt% (MH) and 9.2 wt% (ML) consistently higher than the calculated H_2_O contents of 1.7 wt% (MD), 8.4 wt% (MH) and 8.3 wt% (ML), respectively (Table 7). The reason for this is that late stage serpentinization has increased the LOIs. This illustrates that care has to be taken when measured LOI are used to constrain fluid loss during metamorphic reactions.

A summary of IODP bulk rock measurements of abyssal serpentinites is given in Deschamps et al. (2013). From the average bulk retrieved for harzburgite, a theoretical maximum of 13.8 wt% H_2_O was present prior to olivine formation and 6.6 wt% H_2_O was present after full antigorite + brucite consumption. This suggests a theoretical maximum average loss of 7.2 wt% H_2_O during the antigorite + brucite reaction if no magnetite was present. The minimum release of 3.4 wt% H_2_O in a fully serpentinized depleted harzburgite is calculated if all iron is present in magnetite. In the dunites the range lies between 4.9 and 9.4 wt% H_2_O for Fe-free and FeO_tot_ bulks, respectively. For serpentinized primitive mantle if total iron is FeO then 2.9 wt% H_2_O is released but if the bulk FeO is subtracted talc is formed instead of brucite.

### Implications of the antigorite + brucite reaction on thermodynamic and geodynamic models

In the literature, the antigorite + brucite reaction has been largely neglected in geodynamic models dealing with volatile recycling, partial melting, slab rheology, earth quakes and its significance in the deep water cycle e.g. (Deschamps et al. 2013; Hacker 2008; Peacock 2020; Rüpke et al. 2004; Schmidt and Poli 1998, 2014; Syracuse et al. 2010; van Keken et al. 2011). The reason for this might be grounded in the controversy about how abundantly brucite forms during serpentinization at mid-ocean ridges and in forearc settings and how much brucite there is in ultramafic rocks at the onset of subduction. For example, in the same IODP samples brucite was identified in one study whereas the other did not (Bach et al. 2004; Vils et al. 2008) showing the difficulty to spot this phase. Additionally, a clear set of criteria to identify olivine that formed through the antigorite + brucite reaction were missing.

Published phase diagrams and thermal models of subduction zones considering the serpentinite reactions commonly use bulk serpentinite or peridotite analyses of various compositions (Hacker 2008; Hacker et al. 2003; Schmidt and Poli 1998; van Keken et al. 2011) for modelling the P–T estimates and fluid release. There are several pitfalls that can occur in such calculations: (1) The antigorite + brucite reaction is ignored leading to an overestimation of the amount of water released at the antigorite breakdown. (2) The Tschermak component in antigorite is not considered, resulting in dehydration reactions involving antigorite that are too low in temperature by 30–50 °C (Padrón-Navarta et al. 2013). (3) The divariant field in which olivine and brucite coexist is too large, resulting in a much larger temperature interval for fluid release at the antigorite + brucite reaction (see discussion above). (4) The effect of magnetite on phase relations and fluid release is not taken into consideration. (5) Commonly, a single peridotite composition is chosen and thus the considerable effect of variable bulk composition on the position of the antigorite + brucite reaction as well as on the amount of fluid released is not taken into account.

## Conclusions

A single serpentinite outcrop at the lower Theodulglacier in the Zermatt-Saas unit documents a full orogenic cycle from mantle exhumation, to seafloor alteration, subduction and exhumation. The dehydration reaction of antigorite + brucite to olivine + chlorite + H_2_O is exceptionally well preserved in this outcrop. In undeformed serpentinites, the occurrence of metamorphic olivine can be used to map out the previous distribution of brucite in seafloor-altered mantle rocks. In deformed serpentinites, metamorphic olivine marks the pathways of the escaping fluid in a network of veins and shear zones. Metamorphic olivine is characterized by highly variable Mg#, low Ni, Ca, Ti, Al and high B and Mn contents. Up to 140 µg/g of H_2_O is found exclusively in Si vacancies. The majority of olivine formation and fluid release, occurred within a narrow field < 30 °C between 550 and 600 °C at fore-arc depths of 60–80 km, liberating between 3.4 and 7.2 wt% H_2_O for a depleted, fully serpentinized harzburgitic composition. The large amount of focused fluid released over a small temperature interval might trigger eclogite formation in the overlying meta-basalts, meta-gabbros, metarodingites and metasediments and could be responsible for earthquakes registered in the oceanic lithosphere at fore-arc depths.

## Supplementary information

**Additional file 1: Table S1.** List of samples used in this study corresponding with locations in Fig. 2.

**Additional file 2: Figure S2. a** Sample FA, large olivine_1_ with sulphide inclusions. **b** Sample FA, olivine_2_ domain no sulphides present. **c** Sample Ol_2_, olivine_2_-rich and sulphide absent domain. **d** Sample Ol_2_, partially retrogressed sulphide-rich domain.

**Additional file 3: Table S2.** Full data set of EPMA analyses for all phases.

**Additional file 4: Figure S3.** Left: Sample 14, Ti-chondrodite grain with Atg, Chl, Di, Ol, Mt. Right: Ilmenite grain in sample 14.

**Additional file 5: Figure S4.** Sample FA, the white rectangle indicates the area mapped in Fig. 10. Lower part: Large olivine_1_ single crystal includes magnetite mesh polygons. Upper part: Small polygonal olivine_2_ generation in textural equilibrium with chlorite and Ti-clinohumite.

**Additional file 6: Table S4.** FTIR analyses of the Ti-clinohumite lamellae bearing olivine_1_ generation.

**Additional file 7: Table S3.** Full data set of EPMA analyses used in Figs. 9 and 14 of spinel group minerals from Piccardo and Vissers (2007), Vils et al. (2008), Pirard et al. (2013) and Müntener (1997).

**Additional file 8: Figure S1.** Drone image from the area indicated by the black rectangle in Fig. 2. Sample localities are indicated with rectangles.

## Data Availability

All samples, including thin and thick sections are stored at the Institute of Geological Sciences at the University of Bern, Bern, Switzerland. All data analysed during this study are included in the article and additional Tables S1–S4 and Figures S1–S4.
