# Supplementary figures and images for: The role of the antigorite + brucite to olivine reaction in subducted serpentinites (Zermatt, Switzerland)
Source: Swiss J Geosci. 2020 Oct 26;113(1):16. doi: 10.1186/s00015-020-00368-0 (PMC7588401; doi:10.1186/s00015-020-00368-0)

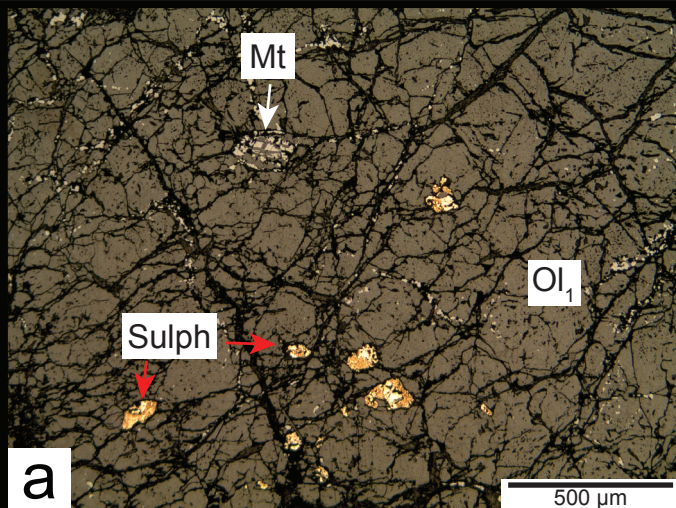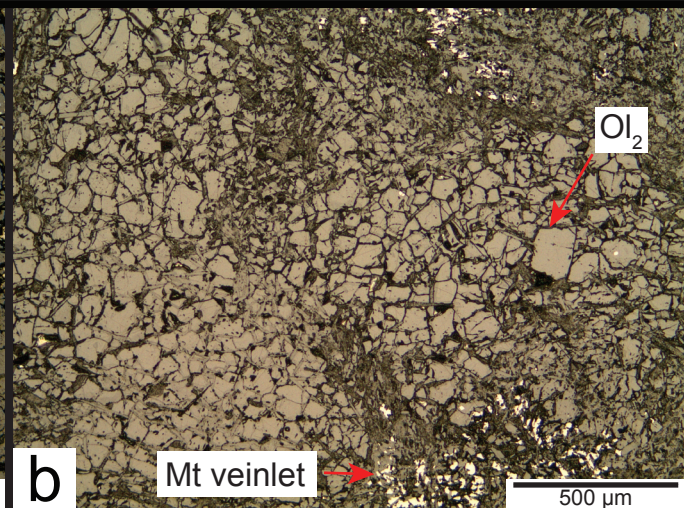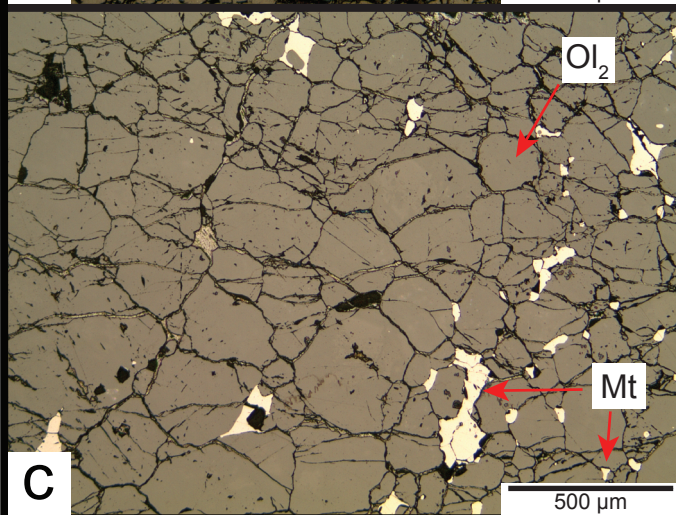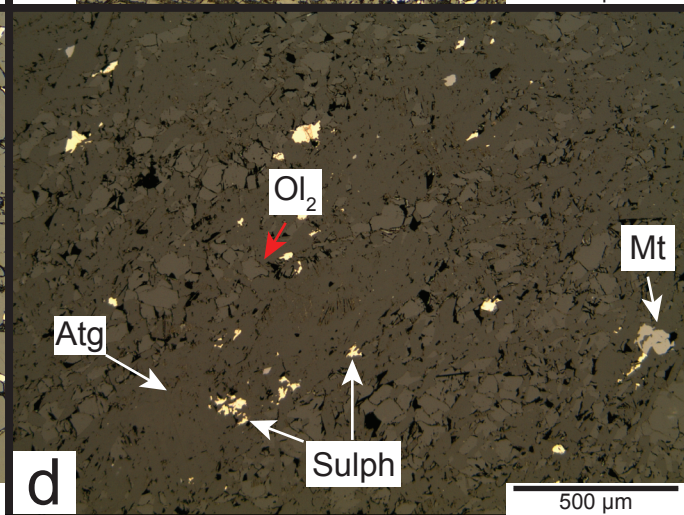

Supplement: Supplementary file 2 — Additional file 2: Figure S2. a Sample FA, large olivine1 with sulphide inclusions. b Sample FA, olivine2 domain no sulphides present. c Sample Ol2, olivine2-rich and sulphide absent domain. d Sample Ol2, partially retrogressed sulphide-rich domain. [file 15_2020_368_MOESM2_ESM.pdf]

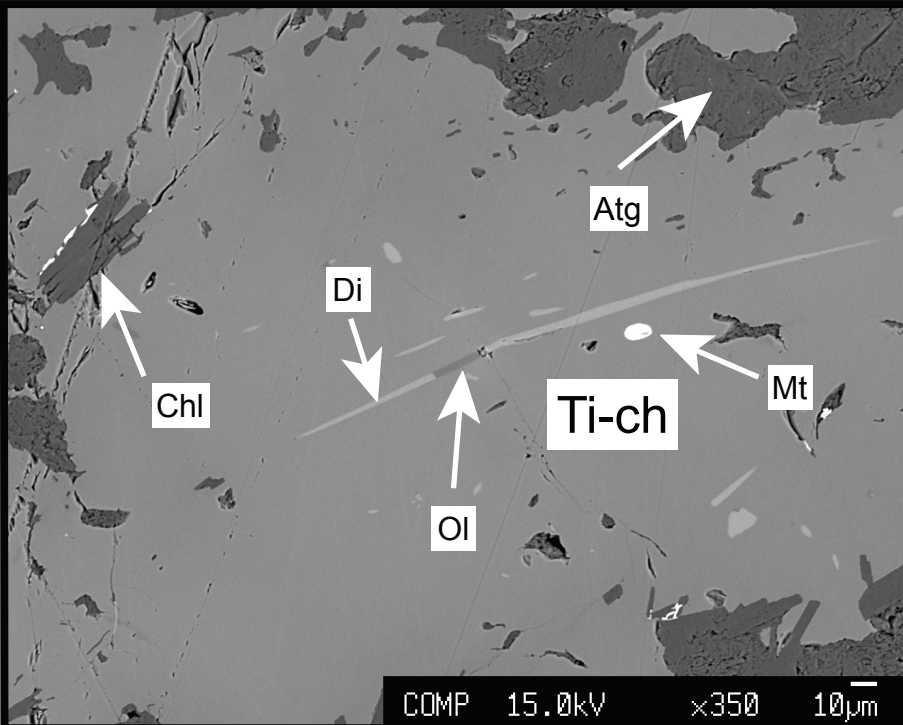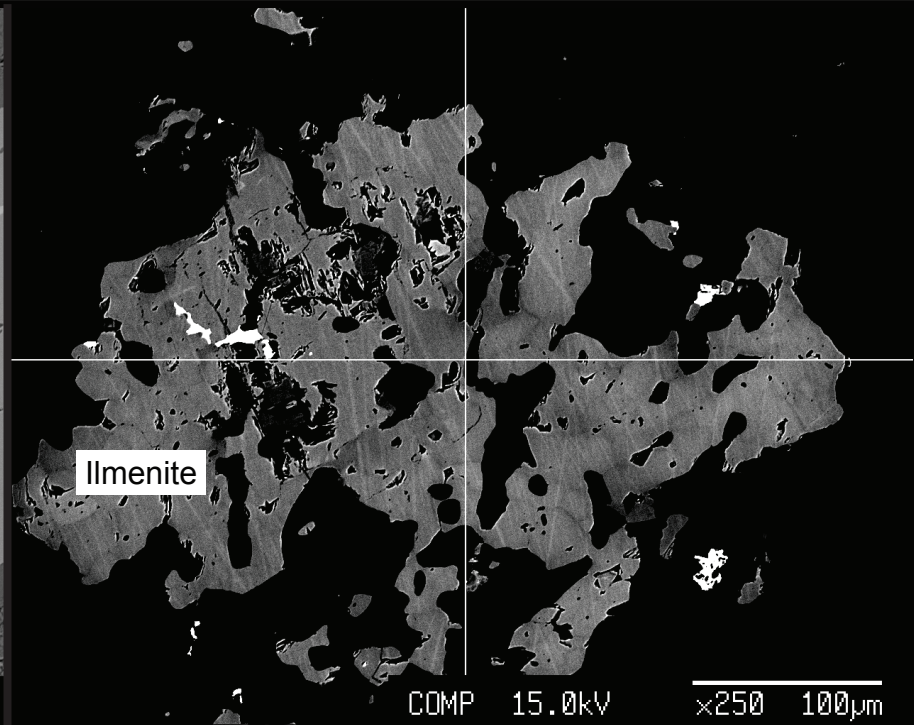

Supplement: Supplementary file 4 — Additional file 4: Figure S3. Left: Sample 14, Ti-chondrodite grain with Atg, Chl, Di, Ol, Mt. Right: Ilmenite grain in sample 14. [file 15_2020_368_MOESM4_ESM.pdf]

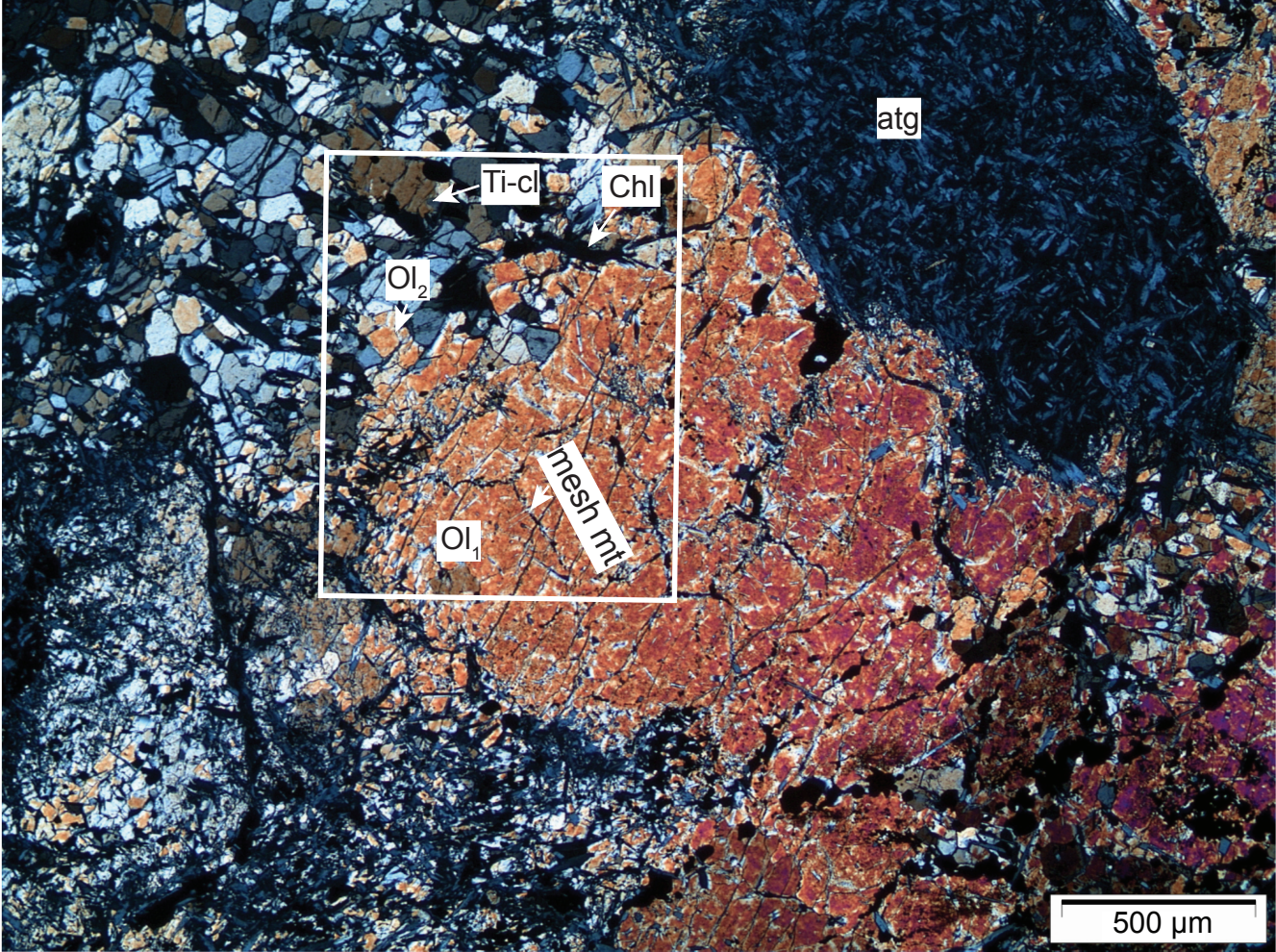

atg

Ti-cl

Chl

Ol<sub>2</sub>

Ol<sub>1</sub>

mesh mt

500  $\mu$ m

Supplement: Supplementary file 5 — Additional file 5: Figure S4. Sample FA, the white rectangle indicates the area mapped in Fig. 10. Lower part: Large olivine1 single crystal includes magnetite mesh polygons. Upper part: Small polygonal olivine2 generation in textural equilibrium with chlorite and Ti-clinohumite. [file 15_2020_368_MOESM5_ESM.pdf]

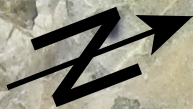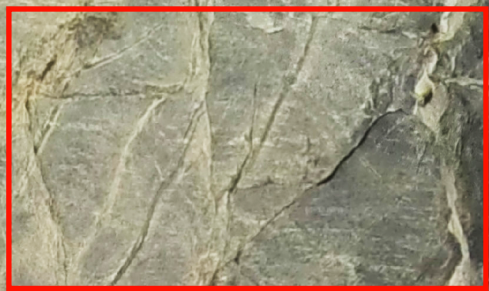

AF2 (7b,c)

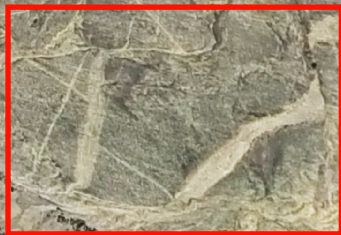

ME (4a,b,e)

10 m

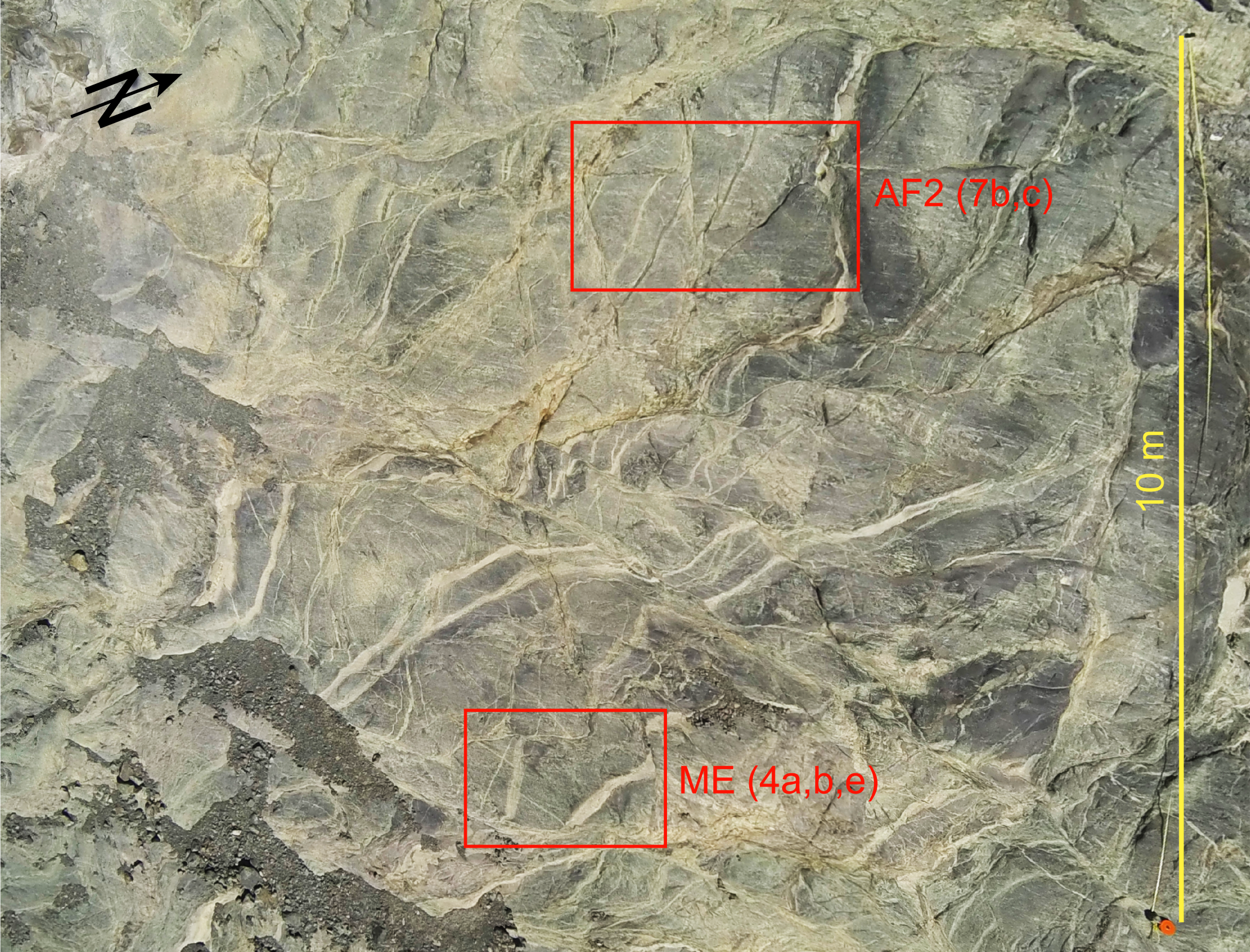

Supplement: Supplementary file 8 — Additional file 8: Figure S1. Drone image from the area indicated by the black rectangle in Fig. 2. Sample localities are indicated with rectangles. [file 15_2020_368_MOESM8_ESM.pdf]
